# Proteoform-Specific Insights into Cellular Proteome Regulation*[Fn FN2]

**DOI:** 10.1074/mcp.O116.058438

**Published:** 2016-07-22

**Authors:** Emma L. Norris, Madeleine J. Headlam, Keyur A. Dave, David D. Smith, Alexander Bukreyev, Toshna Singh, Buddhika A. Jayakody, Keith J. Chappell, Peter L. Collins, Jeffrey J. Gorman

**Affiliations:** From the ‡Protein Discovery Centre and; §Statistics Unit, QIMR Berghofer Medical Research Institute, Herston, Queensland, Australia,; ¶Respiratory Virus Section, Laboratory of Infectious Diseases, National Institute for Allergy and Infectious Diseases, NIH, Bethesda, Maryland, and; ‖School of Chemistry and Molecular Biosciences, The University of Queensland, St Lucia, Queensland, Australia

## Abstract

Knowledge regarding compositions of proteomes at the proteoform level enhances insights into cellular phenotypes. A strategy is described herein for discovery of proteoform-specific information about cellular proteomes. This strategy involved analysis of data obtained by bottom-up mass spectrometry of multiple protein OGE separations on a fraction by fraction basis. The strategy was exemplified using five matched sets of lysates of uninfected and human respiratory syncytial virus-infected A549 cells. Template matching demonstrated that 67.3% of 10475 protein profiles identified focused to narrow pI windows indicative of efficacious focusing. Furthermore, correlation between experimental and theoretical pI gradients indicated reproducible focusing. Based on these observations a proteoform profiling strategy was developed to identify proteoforms, detect proteoform diversity and discover potential proteoform regulation. One component of this strategy involved examination of the focusing profiles for protein groups. A novel concordance analysis facilitated differentiation between proteoforms, including proteoforms generated by alternate splicing and proteolysis. Evaluation of focusing profiles and concordance analysis were applicable to cells from a single and/or multiple biological states. Statistical analyses identified proteoform variation between biological states. Regulation relevant to cellular responses to human respiratory syncytial virus was revealed. Western blotting and Protomap analyses validated the proteoform regulation. Discovery of STAT1, WARS, MX1, and HSPB1 proteoform regulation by human respiratory syncytial virus highlighted the impact of the profiling strategy. Novel truncated proteoforms of MX1 were identified in infected cells and phosphorylation driven regulation of HSPB1 proteoforms was correlated with infection. The proteoform profiling strategy is generally applicable to investigating interactions between viruses and host cells and the analysis of other biological systems.

Gene expression only correlates moderately with protein abundance (1, 2) because of influences such as differential mRNA and protein turnover, regulation of translation, and ubiquitin-mediated proteasomal degradation (3). Consequently, characterization of changes in cellular proteomes is essential to fully understand how cells adapt to intrinsic or extrinsic conditions. Cellular adaptation may involve changes in the global abundances of some proteins. In addition, the proteome may be regulated by generation of different molecular forms of gene products, called proteoforms (4). Proteoforms can arise from alternative splicing, sequence polymorphisms, proteolysis and post-translational modifications (PTMs)^1^. Therefore, characterization of the proteome at both global protein abundance and proteoform levels is essential to fully understand cellular responses.

Advances in both high performance liquid chromatography (HPLC) and mass spectrometry (MS) technologies have greatly facilitated quantification of global protein abundance (5–7). This usually involves bottom-up MS which is characterized by protease digestion of proteins prior to HPLC-tandem mass spectrometry (MS/MS). Peptide sequences are matched to MS/MS spectra and the resultant peptide-to-spectrum matches (PSMs) are used to infer protein identities. Assembling experimentally identified PSMs *in silico* to infer protein identities is a nontrivial task because ambiguities in protein identification arise when peptides match multiple protein sequences. Thus, protein sequences matching the same set or a subset of the same peptide sequences are generally reported together as a protein group (8–13). Computational approaches have been described for detecting different proteoforms in bottom-up MS data (14–16), however, certain proteoforms are unlikely to be distinguished by these methods. For instance, proteoforms that are not expressed in a basal state but are induced in stimulated cells will be difficult to distinguish, particularly if they are also subjected to proteolysis. Proteoform diversity arising from PTMs, such as phosphorylation and proteolysis, can also complicate discrimination between proteoforms. Specific enrichment of modified peptides from protease digests, such as titanium dioxide (TiO_2_) enrichment of phosphopeptides (17), is often required to observe peptides with PTMs. The bottom-up nature of these protocols precludes the assignment of combinations of modifications to specific proteoforms.

Unambiguous identification of splice variants is only possible when peptides exclusively matching the splice form are confidently identified (16). Targeted approaches such as selected reaction monitoring (SRM) (18) can be used to increase the likelihood of discriminating between splice variants by analyzing preselected splice variant specific peptide sequences. However, such peptides may not be experimentally observed. Furthermore, proteoform diversity of splice variants may arise because of PTMs, such as phosphorylation and proteolysis, in regions common to multiple alternate splice variants. Such proteoform diversity will not be reflected through observation of the splice variant specific PSMs. In addition, performance of SRMs for all potential splice forms present in a cell lysate may be prohibitive.

Sequence variants, proteolysis and other PTMs can potentially be differentiated based on unique masses of intact proteins using top-down MS approaches (4, 7, 19). Although recent applications of top-down proteomics identified over 5000 proteoforms (20), currently the top-down approach is considerably more technically demanding than the bottom-up approach (7, 20, 21).

Identification of proteoforms may potentially be achieved through intermediate approaches that involve separation of intact proteins followed by bottom-up MS analysis (7, 22, 23). Intact proteoforms that differ in size may be separated and identified using a workflow called Protomap (22). This involves one-dimensional (1D) SDS-PAGE separation followed by slicing of gel lanes into multiple bands. Each band is subsequently subjected to in-gel digestion and analysis by HPLC-MS/MS. This method is not technically challenging but is restricted to size-based differentiation between proteoforms. Two-dimensional (2D) gel-based IEF coupled with SDS-PAGE may resolve distinct proteoforms, however, this method exhibits bias for certain classes of proteins (24, 25) and the number of proteins identified is often limited (26, 27).

A proteome profiling strategy is described herein that is based on in-solution protein IEF (otherwise known as protein Off-Gel Electrophoresis or herein referred to as protein OGE (28, 29)) for separation of proteoforms in whole cell lysates (5, 30, 31) followed by bottom-up MS (Fig. 1*A*). This approach takes advantage of IEF separation analogous to the first dimension of 2D-IEF/SDS-PAGE but with the benefit that fractionation is performed in a detergent rich denaturing solution phase that is conducive to enhanced recovery of separated proteins.

Human respiratory syncytial virus (hRSV) is a *pneumovirus* from the *pneumovirinae* subfamily of the *paramyxoviridae* family of viruses. Serious lower respiratory tract infection of infants, young children and immunocompromised individuals, including the elderly, can be caused by hRSV infections (32, 33). Previous global transcriptomic, proteomic and biological studies have indicated that hRSV-infected A549 cells endeavor to mount antiviral responses (5, 26, 32, 34). However, hRSV suppresses these antiviral responses by mechanisms that are not completely understood. Investigation of hRSV regulation of the host cell proteome at the proteoform level may provide a more comprehensive understanding of the interaction of hRSV with host antiviral defenses. Thus, potential cellular proteoform regulation by hRSV provided a relevant context for assessment of the proteoform profiling strategy described herein. This involved analysis of a proteomic data set obtained after fractionation of whole cell lysates of uninfected and hRSV-infected A549 cells by protein OGE (5).

The data set examined herein was previously analyzed to identify protein groups with global differences in abundance induced by hRSV infection (5). The current fraction by fraction analysis aimed to determine if proteoform-specific information could be obtained from this data set. Efficacious and reproducible focusing by protein OGE was demonstrated (Fig. 1*B*) and a novel proteoform profiling strategy was developed that successfully identified proteoform regulation induced by hRSV. Proteoforms from some hRSV-induced protein groups were found to be further regulated by alternative splicing and/or by PTMs such as phosphorylation and proteolysis (*e.g.* PSMB10, STAT1, WARS, and MX1). In addition, the heat shock protein Hsp27, which was not regulated at the total protein level, was found to be regulated by phosphorylation. The proteoform profiling strategy described herein is generally applicable to defining cellular responses to other viruses and characterization of other biological systems.

## EXPERIMENTAL PROCEDURES

### 

#### 

##### Data Used for Proteoform Profiling

Data from bottom-up MS analyses of protein OGE separations of hRSV-infected and uninfected A549 cells were acquired and processed as reported in detail previously (5). The data set can be downloaded from the MassIVE repository (massive.ucsd.edu/ProteoSAFe/datasets.jsp) with identifier MSV000079453.

In brief, data from five independent matched replicate sets of uninfected and hRSV-infected type II alveolar lung epithelial A549 cells were used (5). Infection of A549 cells was at a multiplicity of infection (MOI) of three plaque forming units (pfu)/cell using a recombinant clone of hRSV containing the A2 genomic sequence (26, 35). A549 cells used in this study were verified as *Mycoplasma* free. Each of the ten samples was lysed 24 h postinfection and individually subjected to protein OGE using a 24 cm nonlinear pH gradient of 3–11. The resultant 24 fractions were individually subjected to tryptic digestion followed by capillary HPLC interfaced with a hybrid linear-ion-trap (LTQ)/OrbitrapXL mass spectrometer.

As previously reported (5), the 240 .RAW files (Xcalibur, ThremoFisher Scientific) resulting from MS analysis of the 24 fractions from the ten protein OGE separations were processed using MaxQuant 1.3.0.5 (10) (see (5) for the full set of MaxQuant parameters). Andromeda (36) was used to search the complete proteome for *Homo sapiens* and hRSV A2 strains (87,636 canonical and isoform sequences downloaded from www.uniprot.org on 21 February 2013). A posterior error probability threshold of 0.05 and a peptide level false discovery rate threshold of 0.001 were applied to accept confidently identified PSMs. A protein level false discovery rate of 0.01 was applied. Peptide and protein identifications for each fraction in each protein OGE separation were reported separately (*i.e.* fractions belonging the same protein OGE separation were not combined). The MaxQuant assembled protein groups were collapsed such that protein sequences arising from the same gene were reported as a single protein group. UniProt accession numbers identified only by peptides matching more than one protein group were excluded from the analysis. Following this procedure, there was no overlap between the protein groups in terms of peptide sequences, UniProt accession numbers or gene names assigned to the protein groups.

In the current study, the protein groups reported previously (5) were further processed using sequence annotations from the UniProt Knowledgebase (UniProtKB). The UniProtKB sequence status and molecule processing annotations were extracted for all UniProt accession numbers assigned to the protein groups (the complete UniProtKB entries were downloaded from www.uniprot.org on 21 February 2013). The UniProtKB sequence status annotation was used to identify UniProt accession numbers with incomplete protein sequences. That is, UniProt accession numbers with sequence status of “fragment” are incomplete protein sequences because of uncertainty in the gene model (*e.g.* no start and/or stop codon). UniProt accession numbers annotated as fragments were excluded from subsequent analyses. The UniProtKB molecule processing annotation was used to identify processed forms of the protein sequence (*e.g.* the extent of the protein sequence after the removal of a signal or transit peptide or other cleavage events). All annotated forms of the protein sequence (precursor and processed) were retained for subsequent analyses. Therefore, taking the UniProtKB annotations into account, the final protein groups examined in the current study consisted of an exhaustive list of UniProt accession numbers that matched at least one PSM and included all processed forms of the protein sequence and excluded all protein sequences annotated as fragments. Finally, for each confidently identified peptide, the corresponding UniProt accession numbers, protein group identifier, protein OGE experiment, fraction number and the number of PSMs were extracted from the MaxQuant results file “evidence.txt.”

##### Analysis of In-Solution Focusing of Identified Protein Groups

Following protein separation by IEF, proteoforms were expected to be concentrated to OGE fractions according to the protein isoelectric point (pI). To examine the efficacy of focusing achieved by the ten OGE separations, the distribution of the identified protein groups across the 24 OGE fractions was assessed.

An observed focusing profile was defined for each protein group in each protein OGE separation and was equal to the number of PSMs for the protein group in each IEF fraction. The focusing profiles for all protein groups in all protein OGE separations were aligned according to the profile maxima. To examine the efficacy of focusing achieved by protein OGE, four templates representing narrow focusing were specified (Fig. 2*B*–2*E*; lower panels). These templates represented focusing to one OGE fraction, symmetric focusing to three OGE fractions and asymmetric focusing to two OGE fractions, with peaks represented by the vectors (0, 1, 0), (0.5, 1, 0.5), (0.5, 1, 0), and (0, 1, 0.5), respectively. Template matching (37) was applied to identify focusing profiles that matched the narrow focusing templates. A Pearson correlation greater than or equal to 0.95 was applied to accept a match between a narrow focusing template and an experimental focusing profile. Template matching was performed without replacement (*i.e.* if a focusing profile matched one of the templates, it was not tested against any of the other templates). Profiles with maxima less than five PSMs were excluded from the template matching analysis.

##### Prediction of Proteoform Focusing by In-solution Protein IEF

Focusing of proteoform sequences was predicted by mapping calculated pIs to the corresponding OGE fractions using the nonlinear reference gradient (Immobiline DryStrip pH 3–11 nonlinear gradient, GE Healthcare; supplementary Fig. S1*A*). The pI for each proteoform sequence was based on the amino acid sequence and was calculated using Compute Mw/pI (38). The pIs for phosphorylated forms of HSPB1 were calculated using the p*K_a_* values of 2.12 for the first ionisation and 7.21 for the second, as specified at http://www.phosphosite.org (39).

##### Correlation Between Experimental pI and Reference pH Gradients

A comparison of experimental protein focusing against the reference pH gradient was made by overlaying the calculated pIs for selected proteoform sequences observed in each OGE fraction against the reference pH gradient (supplementary Fig. S1*A*). Proteoform sequences were selected based on the following criteria: (1) at least one identified PSM matched the UniProt accession number exclusively; (2) the focusing profile matched one of the narrow focusing templates with a correlation greater than or equal to 0.9; (3) the maximum over the focusing profile was at least five PSMs; and, (4) the UniProtKB entry specified at most one processed proteoform sequence. The pI for each proteoform sequence was calculated using the proteoform sequence after molecular processing (as specified in the UniProtKB entry).

##### Concordance Between Observed and Predicted Protein Focusing

Focusing by protein OGE was predicted for each proteoform sequence using the reference pH gradient and compared with the observed focusing profile for the corresponding protein group. To predict focusing by protein OGE, the calculated pI for each proteoform sequence was mapped to the corresponding OGE fraction using the non-linear reference pH gradient. An error tolerance window was specified around the predicted fraction to allow some prediction error and some minor differences between protein OGE separations. Concordance between the observed focusing profile (*i.e.* distribution of PSMs across the OGE fractions) and predicted focusing for a proteoform sequence was estimated as the proportion of PSMs matching the proteoform sequence within the predicted focusing window, relative to all PSMs matching the protein group. That is, for each proteoform sequence *S_i_* belonging to the protein group, *G* = {*S*_1_, …, S*_n_*}, the concordance between the observed and predicted focusing was estimated as:

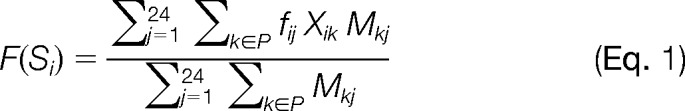


In equation 1, *P* = {k_1_, …, *k_l_*} is the set of peptide sequences assigned to the protein group. *j* is the fraction number, *f_i_* is the predicted fraction for sequence *i* and *f_ij_* is an indicator variable specifying the error tolerance window which is equal to one if (*f_i_* − Δ) ≤ *j* ≤ (*f_i_* + Δ) and is equal to zero otherwise. Here Δ is the error tolerance parameter, where Δ = 1 is applied in all subsequent analyses unless specified otherwise. *X_ik_* is also an indicator variable which is equal to one if the peptide *k* matches a sub-sequence of the proteoform sequence *S_i_* and is equal to zero otherwise. Finally, *M_kj_* is equal to the number of PSMs to the peptide *k* in fraction *j*.

Concordance calculated according to equation 1 is an estimate of how well a single proteoform sequence explains the observed focusing profile for the corresponding protein group. To allow more than one proteoform sequence to contribute to the concordance value (*i.e.* group concordance), equation 1 can be generalized as follows:

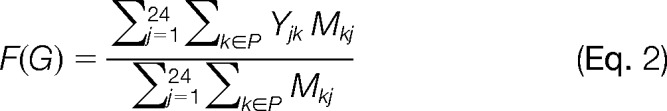
 where *Y_jk_* is an indicator variable that is equal to one if there exists a proteoform sequence *S_i_* predicted to focus to fraction *j* that contains peptide *k* (*i.e.* there exists a sequence *S_i_* belonging to *G* where *f_ij_X_ik_* = 1). *Y_jk_* is equal to zero otherwise.

In this work, the observed focusing profile (*i.e. M* in equation 1 and 2) was represented as the sum over a set of protein OGE separations. That is, for a protein group the number of observed PSMs, *M* for the peptide in fraction *j* is equal to

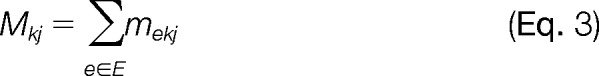
 where *E* is the set of protein OGE experiments, *m_ekj_* is the number of PSMs for the peptide *k* in fraction *j* in OGE separation experiment *e*.

##### Discovery of Proteoform Diversity

Two approaches were applied to identify protein groups with evidence of diversity. The first approach utilized observed focusing profiles and concordance analysis to identify protein groups with evidence of more than one proteoform. Differentially focused proteoforms may be observed as distinct resolved peaks and/or broadening of the observed focusing profile. For the profile analysis, the sum was taken over the ten protein OGE separations according to equation 3. Two values were calculated to describe the observed focusing profile; the number of fractions containing PSMs (*f*) and the width of the focusing profile (*w*, which is equal to the number of fractions from the first OGE fraction containing PSMs to the last OGE fraction containing PSMs, inclusive). The larger the difference between *w* and *f*, the more likely the profile had resolved proteoforms. The group and proteoform concordance values were also used to identify protein groups with evidence of more than one proteoform sequence. A group concordance value that exceeds the largest proteoform concordance is indicative of more than one proteoform sequence. That is, more than one proteoform sequence was necessary to explain a greater proportion of the observed focusing profile.

Redistribution of proteoforms induced by viral infection was assessed using a bootstrapped two-sample Kolmogorov-Smirnov (KSb) test. The nonparametric KSb test was used to assess whether the observed focusing profiles for uninfected and hRSV-infected cells were likely to have come from a common distribution. The KSb test is sensitive to differences in the location and shape of the observed focusing profiles, but the nonparametric test statistic is derived from the empirical data instead of a specified distribution. The KSb test was performed in R (version 3.3.0; (40)) using the ks.boot function from the Matching package (version 4.9–2; (41–43)) with 10,000 simulations to estimate the bootstrap *p* values. The null hypothesis was that the empirical distribution functions of uninfected and hRSV-infected OGE separations were identical, which were tested with the Kolmogorov-Smirnov test statistic. For each protein group, the distributions of PSMs in uninfected and hRSV-infected OGE separations were used as input for ks.boot, where the aggregate sum of each was taken over the five replicate separations. Accordingly, the sample sizes were equal to the total number of PSMs observed in uninfected and hRSV-infected separations, respectively. The KSb test was applied for protein groups that were robustly detected in both uninfected and hRSV-infected lysates that had a profile width, *w*, greater than one OGE fraction. Protein groups were considered robustly detected if at least five PSMs were observed in matching OGE fractions for five uninfected and/or five hRSV-infected separations. Protein groups were ranked according to the bootstrap *p* value, where a small *p* value indicated it was unlikely the observed focusing profiles for uninfected and hRSV-infected cells came from the same distribution. Tied *p* values were ranked according to the KSb test statistic (ordered largest to smallest). The Benjamini-Hochberg adjustment (44) was applied to the bootstrap *p* values to correct for multiple comparisons, using the p.adjust function in R (version 3.3.0).

##### Visualization of Protein Focusing by In-Solution IEF

The focusing of selected proteoform sequences was visualized using sequence coverage heat maps generated with custom scripts written in R (version 2.15.1 (40)). The proteoform sequence coverage was plotted against the OGE fraction using a heat map color scale to represent the total number of PSMs at each position along the sequence. A focusing profile representing the total number of PSMs for the proteoform sequence in each OGE fraction was plotted to the left of the heat map. Peptides matching more than one protein group were not represented in the sequence coverage heat maps or the focusing profile plots.

##### Western Blotting

For 1D-Western blotting, 5 μg of unfractionated A549 lysate or pooled protein OGE fractions were prepared, subjected to 1D-SDS-PAGE and processed as described in detail previously (5). Lysates were prepared using four different biological replicate sample sets of uninfected and hRSV-infected A549 cells to those used for the protein OGE analyses. Cytokine treatments of four biological replicates were performed as described previously (5), except T75 flasks were seeded with 5 million A549 cells prior to cytokine treatments. Primary antibodies to PSMB10 (rabbit; Cat# ab190790), STAT1, (rabbit Cat# ab109320), WARS (rabbit Cat# ab31536), and MxA (rabbit Cat# ab95926) were from Abcam (Cambridge, UK). Monoclonal antibodies to Hsp27 (mouse; Cat # 2402) and specific to the proteoform of Hsp27 phosphorylated on serine82 (Hsp27 pS82) (rabbit; Cat # 9709) were obtained from Cell Signaling Technologies, (Danvers, MA). Primary antibody to β-actin was from Sigma-Aldrich (St Louis, MO). SDS-PAGE gels and PVDF membrane were from Bio-Rad (Hercules, CA). Secondary anti-Rabbit IgG (Donkey), conjugated with IR800CW, and the secondary anti-mouse IgG (Donkey), conjugated with IR680LT, were both from LiCor (LI-COR, Lincoln, NE). Membranes were washed thoroughly and scanned using an Odyssey Infrared Imaging System controlled by Image Studio 4.0 Software (LI-COR) and analyzed quantitatively in Graphpad Prism 6.05 (la Jolla, CA).

Protein samples used for 2D-Western blotting were taken from the four biological replicate sample sets used for 1D-Western blotting described above. Lysates were enriched and quantified as described previously (26) and reconstituted in protein IEF buffer comprising 7 m urea, 2 m thiourea, CHAPS (4% w/v), Ampholytes (0.5% (v/v); pI 3–10 non-linear), dithiothreitol (40 mm) and bromphenol blue (0.002%, (w/v)). Reagents and IEF strips were from GE Healthcare (Little Chalfont, UK), except bromphenol blue, which was from Sigma-Aldrich. Protein samples (20 μg/strip) in IEF buffer were used to rehydrate 7 cm 3–10 non-linear IPTG strips for 12 h at 40 V followed by focusing for 7500 Vh using an Ettan IPGphor3 instrument (GE Healthcare). The focused strips were sequentially incubated for 15 min in reducing and then alkylating SDS equilibration solution. Dithiothreitol (DTT, 50 mg/5 ml) or iodoacetamide (125 mg/5 ml) were freshly added to SDS equilibration solutions for reduction or alkylation, respectively. The SDS equilibration solution was comprised of urea (6 m), Tris-HCl (pH 8.8, 75 mm), glycerol (29% v/v), sodium dodecyl sulfate (SDS, 2% w/v), and bromphenol blue (0.002% w/v). After alkylation, IEF focused proteins were separated in the second dimension by SDS-PAGE using 4–20% miniprotean TGX gels and transferred to PVDF membranes as for 1-D blotting as described previously for 1D-Western blotting (5). Statistical treatment of quantitative data sets (*n* = 4) used methods reported for each experiment. *p* values are reported for comparisons between treatments used in each experiment.

##### Protomap Analysis

Approximately 33 μl (∼11% of the total fraction volume) aliquots of hRSV-infected and uninfected protein OGE fractions five, six, and seven were combined as two separate pools prior to reduction by addition of 1 m DTT to a final concentration of 10 mm and incubation for 16 h at 4 °C and 2 h at 22 °C. The samples were alkylated and concentrated as described previously (26). Approximately one third of the combined and concentrated fractions (five, six, and seven) from hRSV-infected and uninfected samples, described above, were separated by 1D-SDS-PAGE using a 4–20% mini-protean TGX precast polyacrylamide gel (Bio-Rad) according to manufacturer's instructions. The SDS-PAGE lanes of the hRSV-infected and uninfected samples were separately excised and each lane sliced into 52 bands and subjected to in-gel digestion described previously (26, 45). After overnight incubation of the gel slices with trypsin solution, peptides from the gel pieces were extracted three times for 45min at 37 °C with 40 μl of 0.1% (v/v) TFA and final extraction was performed in 5% (v/v) TFA in 50% (v/v) aqueous ACN. The trypsin supernatant and extracts were pooled and completely dried. In addition, ∼15 μg of protein from one of the whole cell lysate sets used for 1D-Western blotting were subjected to 1D-SDS-PAGE and subsequent steps described above. Resultant digests were reconstituted in 10 μl of 0.1% (v/v) TFA in 2%(v/v) aqueous ACN and 5 μl was subjected to nanoUltraHighPressure Chromatography -MS/MS analysis using a Waters (Milford, MA) NanoAcquity UltraHighPressure chromatography system interfaced to an LTQ-Orbitrap VelosPro hybrid mass spectrometer (Thermo Fisher Scientific, Bremen, Germany) as described previously (5, 45). The peptides were separated at 35 °C using a sequence of linear gradients: to 27% B over 45 min; to 40% B over 3 min; and, to 95% B over 4 min followed by 95% B for 5 min. The MS/MS data were processed using Proteome Discoverer (version 1.4.1.14; Thermo Fisher Scientific), and searched with Sequest HT (46) against the same database as the protein OGE data (5). Enzymatic cleavage was set to tryptic (allowing a maximum of two missed cleavages) and carbamidomethylation of cysteine was specified as a fixed modification. Deamidation of asparagine/glutamine and methionine oxidation were specified as variable modifications. Fragment ion and precursor ion mass tolerances were set to 0.8 Da and 20 ppm, respectively. Validation of PSMs was performed using Percolator (47) with a q-value threshold of 0.01. A minimum of two peptides per protein was required for identification and ambiguous protein identifications were reported as protein groups.

##### PCR

A549 cells were infected with the A2 isolate of hRSV at MOI of 1.0 pfu/cell or mock-infected. At 24 h following infection supernatants were removed and mRNA was extracted using Multisource Total RNA Miniprep Kit (Axygen, Union City, CA). This was then used to synthesize cDNA using the Tetro cDNA synthesis kit (Bioline, Alexandria, NSW Australia). PCR with the phusion polymerase (New England Biolabs, Ipswitch, MA) was used to amplify the MxA coding sequence using specific forward (5′-cttactttgcaaagaaggaagatg) and reverse (5′-gtctgctagaaatgagtttattacag) oligonucleotides. PCR was designed to amplify nucleotides 961 to 3426 based upon MxA transcript variant 1 (RefSeq accession number NM_001144925.2) including the complete coding sequence. An identical product of 2465nt would also be produced by the presence of MxA transcript versions 2 and 3 (RefSeq accession numbers NM_002462.4 and NM_001178046.2) whereas a shorter version of 1980nt would be expected for the presence of MxA transcript version 4 (RefSeq accession number NM_001282920.1).

## RESULTS

**Fig. 1.**
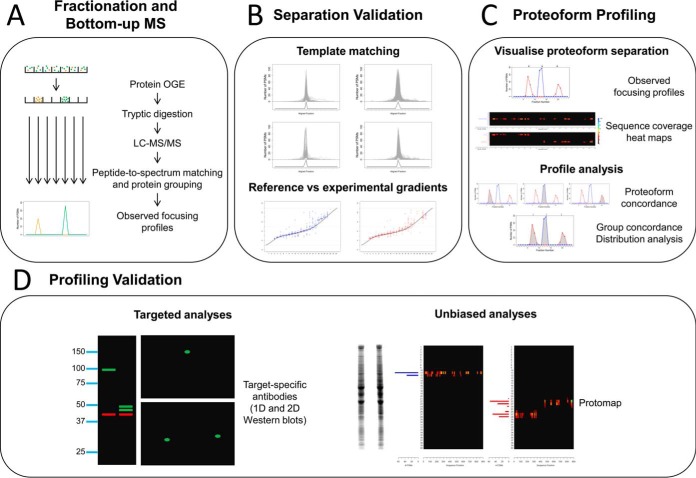
**Proteoform profiling strategy.** *A*, Intact proteoforms are separated into 24 fractions by protein OGE. Each fraction is digested and analyzed by HPLC-MS/MS. The MS/MS spectra are matched to peptide sequences and assembled into protein groups. Observed focusing profiles are extracted for each protein group in each protein OGE separation. *B*, The quality of the protein OGE separations are assessed for evidence of well resolved proteoform focusing (template matching) and consistency with the 3–11 nonlinear reference pH gradient. *C*, Proteoforms may be discovered by identifying protein groups with multiple and/or broad IEF peaks in combination with a novel concordance analysis. Evidence of proteoform redistribution may be detected using the KSb test. *D*, Multiple orthogonal analyses can be applied to validate proteoforms and/or deconvolute complex proteoform profiles. These analyses include: targeted approaches such as 1D- and 2D-Western blotting and unbiased approaches such as Protomap analysis.

The findings reported herein were derived from the reanalysis of a previously published protein OGE data set (5). The data set was generated using bottom-up analysis of ten protein OGE separations (Fig. 1*A*). MaxQuant analysis identified 3412 protein groups and a total of 521,801 PSMs corresponding to 28,895 peptide sequences with distinct amino acid composition (473,567 PSMs after removing shared peptides). Label free quantification was previously used to identify global changes in protein abundance in response to hRSV infection, where the 24 OGE fractions were combined to quantify proteins at the sample level. One-hundred and fourteen protein groups were identified as having significant differences in abundance between uninfected and hRSV-infected cells (FDR < 1%). The majority of regulated cellular proteins were up-regulated and were consistent with induction of IFN responses in the host cell by hRSV. Although the global analysis identified protein groups and cellular pathways affected by hRSV infection, insight into regulation of specific proteoforms was not apparent in this global approach. Thus, the distribution of protein groups across the OGE fractions was interrogated in the current study to identify differentially abundant proteoforms.

### 

#### 

##### Efficacy of In-Solution Protein IEF

Two approaches were taken herein to assess the efficacy and reproducibility of the protein OGE process (Fig. 1*B*). The first approach addressed efficacy by examining whether focusing by protein OGE was well resolved (*i.e.* whether protein species focused to narrow regions of the pH gradient). The second approach addressed reproducibility by examining consistency of migration of protein species relative to the 3–11 nonlinear reference pH gradient used for separation.

**Fig. 2.**
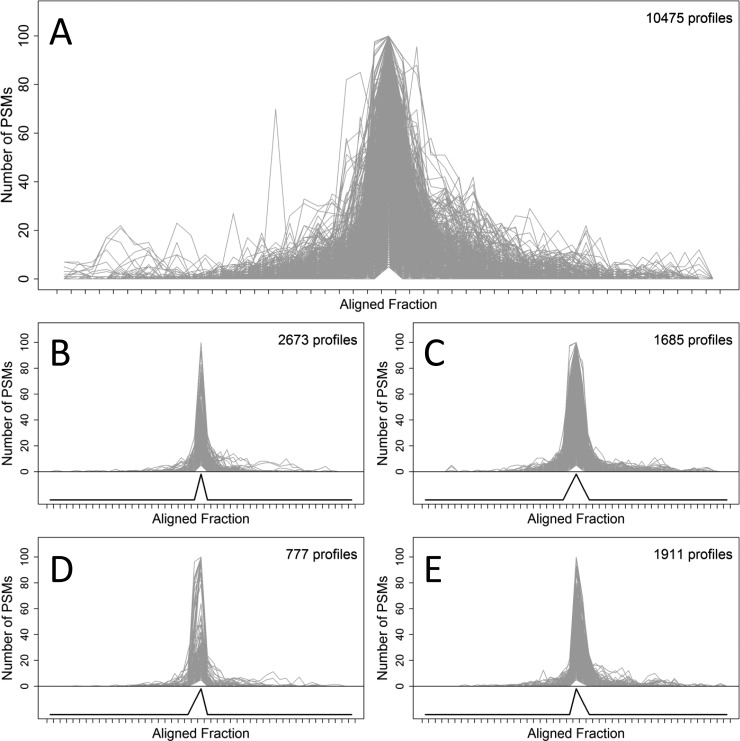
**Template matching of in-solution protein IEF focusing profiles.** Aligned focusing profiles for: *A*, 10475 profiles that were subjected to template matching; *B*, 2673 profiles that matched the template representing focusing to a single OGE fraction; *C*, 1685 profiles that matched the template representing symmetric focusing to three OGE fractions; *D* and *E*, profiles that matched the templates representing asymmetric focusing to two OGE fractions (777 and 1911 profiles, respectively). The aligned focusing profiles are represented as gray lines and are equal to the number of PSMs for a protein group in each fraction. The templates are represented as a black line below the aligned focusing profiles in panels *B–E*. A Pearson correlation greater than or equal to 0.95 was applied to accept a match between a narrow focusing template and an experimental focusing profile. Profiles with maxima greater than 100 PSMs were rescaled for plotting.

In the first approach, the distribution of the identified protein groups across the 24 OGE fractions of each protein OGE separation was examined. A focusing profile was defined for each protein group in each protein OGE separation, which was comprised of the number of observed PSMs for the protein group in each OGE fraction. Because ten protein OGE separations were performed, up to ten focusing profiles were obtained for each protein group. A total of 23,936 nonzero focusing profiles were obtained for the 3412 protein groups identified in the previous global analysis (5). After excluding 13,461 profiles with maxima less than five PSMs, the focusing properties of 10,475 focusing profiles (Fig. 2*A*) were examined using template matching. The majority of the profiles (7046 or ∼67.3%) conformed to at least one of four narrow focusing templates, with a correlation greater than or equal to 0.95 (Figs. 2*B*–2*E*). These templates represented focusing to a single OGE fraction (Fig. 2*B*), symmetric focusing to three OGE fractions (Fig. 2*C*) and asymmetric focusing to two OGE fractions (Figs. 2*D* and 2*E*), which matched 2673, 1685, and 777 or 1911 profiles, respectively. Thus, it was apparent that a generally high level of efficacy was achieved in the protein OGE process.

In the second approach, the calculated pIs for proteoform sequences observed to focus to each OGE fraction were compared with the expected distribution according to the reference pH gradient. To achieve this, the distribution of experimental pI values for each OGE fraction was determined using the calculated pI values for proteoform sequences selected according to the criteria prescribed in the Experimental Procedures above. For all protein OGE separations there was very good agreement between the reference pH and experimental pI gradients (supplementary Fig. S1), except for minor deviations observed in the basic pI region (from fraction 18 to 22). In addition, insufficient protein species were observed to focus to the end fractions 1, 2, 23, and 24 to assess focusing at the very acidic and basic ends of the gradients. In combination, these results demonstrate the achievement of highly efficacious and reproducible protein OGE.

##### Concordance Analysis

Given the apparent efficacy and reproducibility of the protein OGE separations demonstrated above, the concordance between the observed focusing profiles and predicted proteoform focusing was assessed (supplementary Table S2). Only proteoforms generated by alternative splicing, alternate translation start sites and proteolytic cleavages specified in UniProt were considered, unless specified otherwise.

Focusing was predicted by mapping the calculated proteoform pIs to the corresponding OGE fractions using the nonlinear reference pH gradient. A focusing window of three OGE fractions was specified to allow for some prediction error and minor differences between the protein OGE separations. The fit between the observed PSMs and the predicted focusing for each proteoform sequence was estimated according to the concordance calculation specified by equation 1. Concordance values are in the range from zero to one. Whereas a proteoform concordance value of zero means the proteoform sequence does not explain any of the observed PSMs, a proteoform concordance value of one implies the proteoform sequence was sufficient to explain all of the observed PSMs for the protein group. A proteoform concordance value between zero and one implies that the proteoform sequence can explain only a portion of the observed PSMs, either because the proteoform sequence does not contain all of the peptide sequences belonging to the protein group and/or the observed focusing profile is not contained within the predicted focusing window (*i.e.* is not aligned with the predicted focusing position or is broader than three IEF fractions).

**Fig. 3.**
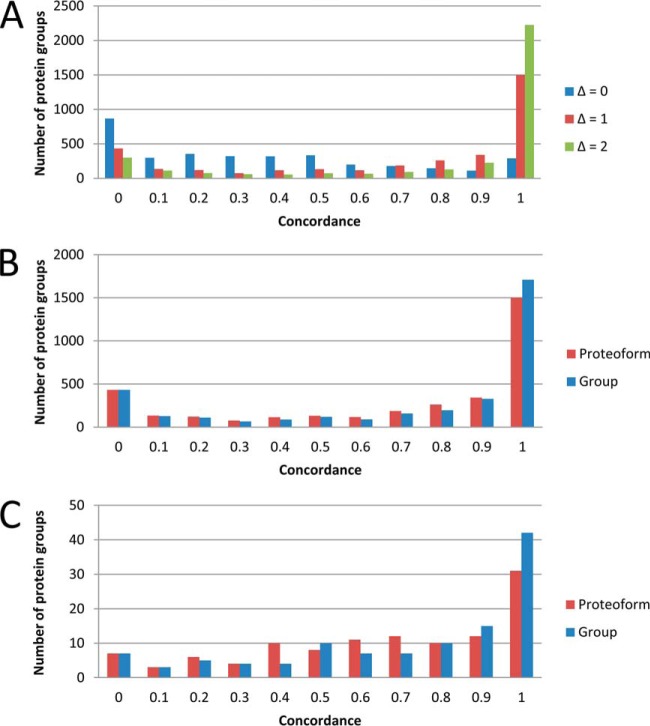
**Protein group concordance distributions.** Proteoform concordance values for *A*, all 3412 protein groups plotted using three different error tolerance windows corresponding to one (Δ = 0), three (Δ = 1) or five (Δ = 2) OGE fractions, respectively. Concordance was calculated according to equation 1 and each protein group was represented by the largest concordance value for an individual proteoform sequence belonging to the protein group. Proteoform (red) and protein group (blue) concordance values for *B*, 3412 protein groups and *C*, the 114 protein groups identified as differentially abundant between uninfected and hRSV-infected A549 cells. The proteoform concordance values presented in red represent the largest portion of the observed focusing profile that can be explained by an individual proteoform sequence belonging to the protein group (equation 1), whereas the group concordance values presented in blue represent the proportion of the observed focusing profile that can be explained by all proteoform sequences belonging to the protein group (equation 2). An error tolerance window of three OGE fractions (*i.e.* Δ = 1) was specified. Note that the concordance bins are for the range (bin_i-1_, bin_i_).

**Fig. 4.**
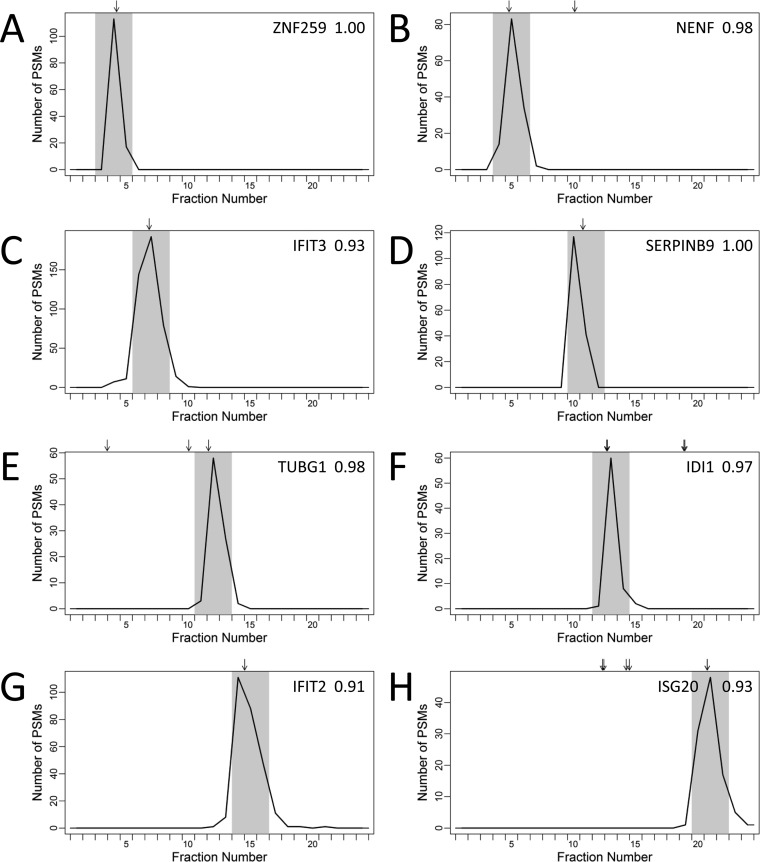
**Focusing profiles for selected protein groups explained by an individual proteoform sequence.** Observed focusing profiles for *A*, ZNF259 (O75312); *B*, NENF (Q9UMX5 32–172); *C*, IFIT3 (O14879); *D*, SERPINB9 (P50453); *E*, TUBG1 (P23258); *F*, IDI1 (Q13907); *G*, IFIT2 (P09913); and, *H*, ISG20 (Q96AZ6) protein groups. Each profile is represented by a black line and is equal to the number of PSMs for the protein group in each OGE fraction, where the sum is taken over the ten protein OGE separations of uninfected and hRSV-infected A549 cell lysates. Arrows represent the predicted focusing for all proteoform sequences belonging to the protein group. The gray shaded regions represent the predicted focusing windows for the proteoform sequences that explain the observed focusing profiles and the proteoform concordance values are presented in the top right corner of the plot (Δ = 1).

The distribution of proteoform concordance values for all protein groups at three different error tolerance windows is presented in Fig. 3*A*. Each protein group is represented by the largest concordance value for an individual proteoform sequence belonging to the protein group. Allowing an error tolerance window of three IEF fractions (*i.e.* Δ = 1), 1501 protein groups had at least one proteoform sequence that had a concordance value greater than 0.9 (*i.e.* 1501 protein groups had an individual proteoform sequence that was sufficient to explain greater than 90% of the observed focusing profile for the protein group). Examples of protein groups with narrow observed focusing profiles that could be explained by a single proteoform sequence included ZNF259, NENF, IFIT3, SERPINB9, TUBG1, IDI1, IFIT2, and ISG20 (Figs. 4*A*–4*H*).

**Fig. 5.**
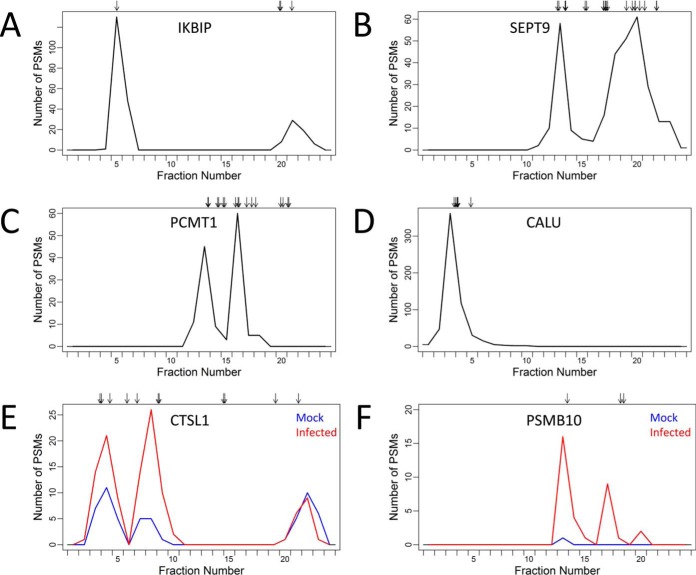
**Focusing profiles for selected protein groups explained by more than one proteoform sequence.** Observed focusing profiles for *A*, IKBIP; *B*, SEPT9; *C*, PCMT1; and, *D*, CALU protein groups. Each profile is represented by a black line and is equal to the number of PSMs for the protein group in each OGE fraction, where the sum is taken over the ten protein OGE separations of uninfected and hRSV-infected A549 cell lysates. Observed focusing profiles for *E*, CTSL1 and *F*, PSMB10 in uninfected and hRSV-infected A549 cell lysates are represented in blue and red lines, respectively, where the sum is taken over the five protein OGE separations of each. Arrows represent the predicted focusing for all proteoform sequences belonging to the protein groups.

The proteoform level concordance values presented in Fig. 3*A* represent how well individual proteoform sequences explained the observed focusing profiles. However, this did not allow for the possibility that more than one proteoform sequence contributed to the observed focusing profile. Allowing more than one proteoform sequence to contribute to the concordance calculation (equation 2), the distribution of concordance values at the group level is presented in Fig. 3*B*. Applying an error tolerance window of three OGE fractions (*i.e.* Δ = 1), 1708 protein groups had a group concordance value greater than 0.9 (*i.e.* greater than 90% of the observed focusing profile for 1708 protein groups could be explained). Furthermore, taking the sum over the 3412 protein groups, a total of 323,785 PSMs were explained by the group concordance analysis, which corresponds to 68% of the PSMs identified by the MaxQuant analysis (where PSMs for shared peptides were excluded). Comparison of the group and proteoform concordance values can be used to discover protein groups with evidence of more than proteoform sequence. That is, a group concordance value exceeding the individual proteoform concordance values indicates that more than one proteoform sequence is necessary to explain a greater proportion of the observed focusing profile. Examples of protein groups with evidence of more than one proteoform sequence included IKBIP, SEPT9, PCMT1, and CALU (Figs. 5*A*–5*D*).

IKBIP is an example of a protein group that had evidence of multiple proteoforms resolved by both pI and exclusive peptide matches (Fig. 5*A*). Peptides exclusively matching proteoform sequence Q70UQ0–4 of IKBIP were observed in the IEF peak at fraction five, which was consistent with the predicted focusing. Similarly, the observed peptides and predicted focusing for the proteoform sequence Q70UQ0 of IKBIP was sufficient to explain the IEF peak at fraction 21. SEPT9 and PCMT1 are examples of protein groups with evidence of multiple proteoforms resolved by pI (Figs. 5*B* and 5*C*). Two resolved IEF peaks were apparent in the observed focusing profile for the SEPT9 protein group. The peak at fraction 13 matched the proteoform sequence Q9UHD8–3 whereas the broad IEF peak spanning fractions 16 to 22 could be explained by the predicted focusing of Q9UHD8, Q9UHD8–2, Q9UHD8–5, and Q9UHD8–7 proteoforms. For the PCMT1 protein group, two proteoform sequences were sufficient to explain the two IEF peaks evident in the observed focusing profile; proteoform sequence P22061–2 matched the IEF peak at fraction 13 and proteoform sequence P22061 matched the IEF peak at fraction 16. The multipeak focusing profiles of these protein groups also indicate proteoform diversity (Fig. 5*A*–5*C*).

For several protein groups the predicted focusing of proteoform sequences was not well resolved by isoelectric point, however, more than one proteoform sequence could be confidently identified by exclusive peptide matches. For example, the eight proteoform sequences belonging to the CALU protein group (Fig. 5*D*) were predicted to focus to fraction three, four or five. However, peptide sequences that matched only the O43852–2 and O43852–4 proteoform sequences were identified in fraction three and peptide sequences that matched only the O43852 and O43852–3 proteoform sequences were identified in fractions three and four. Therefore at least two proteoform sequences are necessary to explain the narrow focusing profile for the CALU protein group.

##### Proteoform Regulation Within Protein Groups Differentially Regulated on a Global Level by hRSV Infection

The 114 protein groups previously identified as differentially abundant between uninfected and hRSV-infected A549 cells (5) were examined for evidence of proteoform regulation. Concordance analysis (Fig. 3*C*) revealed that 31 of the hRSV regulated protein groups, a single proteoform sequence had a concordance of greater than 0.9 (*e.g.* IFIT3, SERPINB9, IFIT2, and ISG20; Fig. 4). In contrast, more than one proteoform sequence was necessary to explain the observed focusing profiles for protein groups including PSMB10, CTSL1, STAT1, WARS, and MX1 (supplementary Table S2). That is, by allowing more than one proteoform sequence to contribute to the concordance value, a greater proportion of the observed focusing profiles were explained (with increases in concordance of 0.29, 0.53, 0.12, 0.13, and 0.23, respectively).

The observed focusing profile for the CTSL1 protein group had evidence of hRSV regulation at the proteoform level. Three resolved peaks were evident for the CTSL1 protein group (Fig. 5*E*). The two acidic peaks appeared to be more abundant in hRSV-infected lysates compared with uninfected lysates. For the CTSL1 observed focusing profile, the IEF peak at fraction eight was consistent with the precursor proteoform sequence (P07711) and the IEF peak at fraction 22 was consistent with the cleaved form (P07711 292–333) called the Cathepsin L1 light chain. The IEF peak at fraction four matched the cleaved form (P07711 114–288, called the Cathepsin L1 heavy chain) or an intermediate cleavage form (P07711 114–333).

For the PSMB10 protein group there was evidence of two resolved peaks in the observed focusing profile, with apparent induction of both by hRSV (Fig. 5*F* and supplementary Fig. S2*A*). The observed focusing was consistent with the predicted focusing for the full length precursor proteoform sequence (P40306, IEF peak at fraction 17) and the activated proteoform sequence (P40306 40–273, IEF peak at fraction 13). The concordance analysis was supported by 1D- and 2D-Western blotting. Species at ∼29 kDa and 25 kDa were observed on 1D-Western blots of hRSV-infected whole cell lysates (supplementary Fig. S3*A*), which are consistent with the full length and activated proteoforms of PSMB10 respectively. No PSMB10 immunoreactivity was detected on 1D-Western blots of uninfected cells (supplementary Fig. S3*A*). Substantial levels of molecular species were observed on 2D-Western blots of infected cell lysates consistent with the full-length and activated proteoforms of PSMB10 (supplementary Fig. S3*B*). An additional very faint species was evident on the 2D-Western blot of the infected cell lysate with the same size as the activated proteoform but at a more acidic pI (supplementary Fig. S3*B*). This is potentially a minor proportion of the activated proteoform that was subjected to an acidic PTM but was not detected in the protein OGE. The 2D-Western blot of the uninfected cells showed a minor level of the activated proteoform (supplementary Fig. S3*B*) that was consistent with the PSMs observed in protein OGE fraction 13 (Fig. 5*F*).

**Fig. 6.**
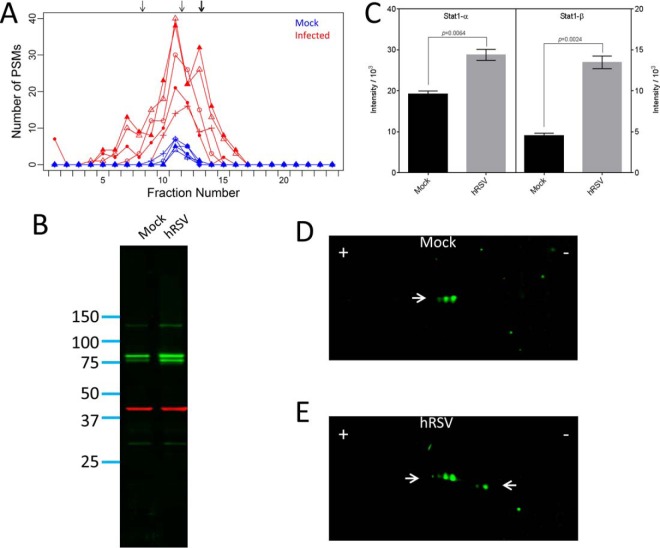
**Analysis of STAT1 proteoforms induced by hRSV infection of A549 cells.** Panel *A* presents the observed focusing profile for the STAT1 protein group. Focusing for the five uninfected lysates are represented in blue and focusing for the five hRSV-infected lysates are represented in red. Arrows represent the predicted focusing for all proteoform sequences belonging to the STAT1 protein group. Panels *B* and *C* present data obtained from 1D-Western blots of four independent biological replicate sets of uninfected (Mock) and hRSV-infected (hRSV) A549 cell lysates. Panel *B*, is a representative image of a 1D-Western blot obtained with equal quantities of protein from Mock and hRSV lysates from one replicate. Stat1-α and –β are evident as green bands with mobilities corresponding to 91 and 84 kDa, respectively. The red band represents β-actin used as a loading control. Quantitative data are presented in panel *C* for data obtained from the complete sample set with the Stat1-α and –β proteoforms presented on the left and right, respectively. Integrated intensities are presented as the mean ± S.E. (*n* = 4). Statistical analysis was performed using a paired, two-tailed Student's *t* test. *p* values are presented in both panels. Western blots after 2D-separation of equal quantities of protein from one replicate of *D* Mock and *E* hRSV lysates. Arrows pointing to the right indicate different proteoforms of Stat1-α and to the left indicate different proteoforms of Stat1-β.

A basal level of STAT1 was observed in the protein OGE separations for the uninfected cell lysates (Fig. 6*A*). For the hRSV-infected lysates, an increase in abundance of STAT1 and broadening of the observed focusing profile was apparent compared with the observed focusing in the uninfected lysates (Fig. 6*A*). The observed focusing profile for the uninfected lysates was consistent with the predicted focusing of the full length Stat1-α proteoform sequence (P42224, concordance of 0.9; Table I). The Stat1-β proteoform sequences (P42224–2 and J3KPM9) could also have contributed to the observed focusing profile (concordance of 0.31 for both Stat1-β proteoform sequences). However, no peptides exclusively matched Stat1-β in the uninfected lysates (supplementary Fig. S2*B*) and the predicted focusing of Stat1-α and Stat1-β proteoforms was not resolved by protein OGE, hence Stat1-β was not conclusively identified in the uninfected lysates.

**Table I:** Concordance values for all proteoform sequences belonging to the STAT1, WARS, MX1 and HSPB1 protein groups

UniProt accession number^a^	Proteoform name	Gene name	Length	MW (kDa)	pI	Predicted focusing window	Proteoform concordance^b^			Nterm^c^	Cterm^c^
							Mock	hRSV	ALL		
P42224 (2–750)	Signal transducer and activator of transcription 1-alpha (STAT1-alpha)	STAT1	749	87.20	5.74	11–13	0.90	0.57	0.59	Y	Y
P42224–2 (2–712)	Signal transducer and activator of transcription 1-beta (STAT1-beta)	STAT1	711	82.91	6.02	12–14	0.31	0.36	0.35	Y	Y
J3KPM9 (2–714)	Signal transducer and activator of transcription 1-beta	STAT1	713	83.23	6.03	12–14	0.31	0.35	0.34	N	Y
D2KFR9 (2–193)	Signal transducer and activator of transcription 1-alpha	STAT1	192	23.02	5.27	7–9	0	0.06	0.05	Y	N
Group		STAT1					0.90	0.69	0.71		
P23381 (2–471)	Tryptophan–tRNA ligase, cytoplasmic (TrpRS)	WARS	470	53.03	5.83	11–13	0.89	0.73	0.76	Y	Y
P23381–2 (2–424)	Isoform 2 of Tryptophan–tRNA ligase, cytoplasmic (mini TrpRS)	WARS	423	48.05	5.91	12–14	0.68	0.58	0.60	N	Y
P23381 (71–471)	T1-TrpRS	WARS	401	45.84	6.02	12–14	0.68	0.57	0.59	N^d^	Y
P23381 (94–471)	T2-TrpRS	WARS	378	43.33	7.12	16–18	0	0.03	0.02	N^d^	N
G3V4A3 (2–36)	T1-TrpRS	WARS	35	3.64	8.90	19–21	0.01	0.01	0.01	Y	N
G3V4Q0 (2–40)	T1-TrpRS	WARS	39	4.22	9.72	21–23	0.01	0.01	0.01	Y	N
Group		WARS					0.95	0.87	0.89		
P20591	Interferon-induced GTP-binding protein Mx1	MX1	662	75.52	5.6	10–12	-	0.63	0.63	Y	N
P20591 (2–662)	Interferon-induced GTP-binding protein Mx1	MX1	661	75.39	5.6	10–12	-	0.63	0.63	Y	N
F8W8T1	Interferon-induced GTP-binding protein Mx1, N-terminally processed	MX1	639	73.34	5.59	10–12	-	0.54	0.54	Y	N
F8W8T1 (2–639)	Interferon-induced GTP-binding protein Mx1, N-terminally processed	MX1	638	73.21	5.59	10–12	-	0.54	0.54	Y	N
P20591–2 (2–508)	Interferon-induced GTP-binding protein Mx1 (varMxA)	MX1	507	55.53	5.00	5–7	-	0.21	0.21	Y	N
P20591–2	Interferon-induced GTP-binding protein Mx1 (varMxA)	MX1	508	55.66	5.00	5–7	-	0.21	0.21	Y	N
P20592	Interferon-induced GTP-binding protein Mx2	MX2	715	82.09	8.91	19–21	-	0	0	N	N
B7Z5D3	Interferon-induced GTP-binding protein Mx2	MX2	201	22.11	8.73	19–21	-	0	0	N	N
Group		MX1					-	0.86	0.86		
P04792 (1–205)	Heat shock protein beta-1	HSPB1	205	22.78	5.98	12–14	0.71	0.48	0.59	N	N
F8WE04 (1–186)	Heat shock protein beta-1	HSPB1	186	20.41	9.25	20–22	0.01	0	0.01	N	N
C9J3N8 (1–37)	Heat shock protein beta-1	HSPB1	37	3.89	5.00	5–7	0	0.01	0.01	N	
Group		HSPB1					0.72	0.49	0.60		

UniProt accession number^a^	Proteoform name	Gene name	No. Phospho	Length	MW (kDa)	pI	Predicted focusing window	Proteoform concordance^b^			Nterm^c^	Cterm^c^
								Mock	hRSV	ALL		
P04792	Heat shock protein beta-1	HSPB1	1	205	22.78	5.75	11–13	0.70	0.51	0.60	N	N
P04792	Heat shock protein beta-1	HSPB1	0	205	22.78	5.98	12–14	0.71	0.48	0.59	N	N
P04792	Heat shock protein beta-1	HSPB1	2	205	22.78	5.56	9–11	0.20	0.40	0.30	N	N
P04792	Heat shock protein beta-1	HSPB1	3	205	22.78	5.39	8–10	0.13	0.33	0.23	N	N
F8WE04	Heat shock protein beta-1	HSPB1	3	186	20.41	6.87	15–17	0.04	0.04	0.04	N	N
F8WE04	Heat shock protein beta-1	HSPB1	2	186	20.41	7.45	17–19	0.01	0.01	0.01	N	N
F8WE04	Heat shock protein beta-1	HSPB1	0	186	20.41	9.25	20–22	0.01	0	0.01	N	N
F8WE04	Heat shock protein beta-1	HSPB1	1	186	20.41	8.57	19–21	0.01	0	0.01	N	N
C9J3N8	Heat shock protein beta-1	HSPB1	0	37	3.89	5	5–7	0	0.01	0	N	N
C9J3N8	Heat shock protein beta-1	HSPB1	1	37	3.89	4.58	3–5	0	0	0	N	N
C9J3N8	Heat shock protein beta-1	HSPB1	2	37	3.89	4.26	2–4	0	0	0	N	N
C9J3N8	Heat shock protein beta-1	HSPB1	3	37	3.89	3.92	1–3	0	0	0	N	N
Group (allowing no phosphorylations)								0.72	0.49	0.60		
Group (allowing up to one phosphorylation)								0.80	0.60	0.70		
Group (allowing up to two phosphorylations)								0.92	0.89	0.91		
Group (allowing up to three phosphorylations)								0.96	0.97	0.96		

^a^Numbers in parentheses represent the portion of the full length UniProt sequence entry represented by the proteoform sequence.

^b^Proteoform concordance values (Δ = 1) for the five uninfected experiments only (Mock), the five hRSV-infected experiments only (hRSV) and the ten IEF experiments combined (ALL).

^c^Indicates whether the N-terminal/C-terminal peptide was identified in a fraction within the predicted focusing window.

^d^N-terminal peptide arising from processing of the canonical sequence was not in the search space (i.e., whereas the processed N-terminal peptides is a semi-tryptic peptide, PSMs were based on trypsin/P searches).

For the hRSV-infected lysates, the predicted focusing for the four proteoform sequences belonging to the STAT1 protein group was sufficient to explain a substantial proportion of the observed focusing profile (group concordance of 0.69; Table I). The proteoform sequences with predicted focusing consistent with observed PSMs were full length Stat1-α (P42224), an unreviewed truncated form of Stat1-α (D2KFR9), the full length Stat1-β (P42224–2), and an unreviewed Stat1-β proteoform sequence (J3KPM9). Peptides exclusively matching Stat1-α (P42224) were identified in fractions five to sixteen. Similarly, PSMs exclusively matching Stat1-β (*i.e.* matching the C-terminal tryptic peptide) were identified in fractions 12 and 13 (supplementary Fig. S2*B*) which is consistent with the predicted focusing for the Stat1-β proteoform sequences (P42224–2 and J3KPM9; supplementary Fig. S2*B*). No peptides exclusively matched the D2KFR9 proteoform sequence. In addition, the D2KFR9 proteoform sequence was not sufficient to explain all the identified PSMs in the predicted focusing window, thus D2KFR9 was not conclusively identified in the hRSV-infected lysates.

The observed peptides and predicted focusing for the STAT1 protein group suggested hRSV-infected A549 cells contained a mixture of Stat1-α and Stat1-β proteoforms. However, the combination of STAT1 proteoform sequences was not sufficient to explain the broad IEF focusing profile in the hRSV-infected lysates. Because a substantial proportion of the observed focusing profile was not explained, STAT1 proteoforms were further examined using 1D- and 2D-Western blotting. Two bands of STAT1 immunoreactivity consistent with Stat1-α (91kDa) and Stat1-β (84 kDa) were observed by 1D- Western blot analysis (Fig. 6*B*). Immunoreactivity of greater than 100 kDa was also observed which does not correspond to any STAT1 proteoform sequence in UniProt. Quantification revealed that both Stat1-α and Stat1-β were induced upon infection with the induction of Stat1-β being greater than for Stat1-α (Fig. 6*C*).

Regulation of STAT1 proteoforms by hRSV-infection was also revealed by 2D- Western blotting (Figs. 6*D* and 6E). Stat1-α was evident as two major spots and a minor spot in uninfected cells apparently reflecting a series of PTMs, such as phosphorylation, but Stat1-β was not observed on these blots (Fig. 6*D*). Both Stat1-α and Stat1-β were observed as multiple spots in hRSV-infected A549 cells (Fig. 6E). The multi-spot distribution of the STAT1 proteoforms is indicative of more extensive PTM of Stat1-α and Stat1-β in infected cells which may contribute to broadening of the focusing profile of the STAT1 protein group for the infected cell lysates relative to the uninfected cells (Fig. 6A).

**Fig. 7.**
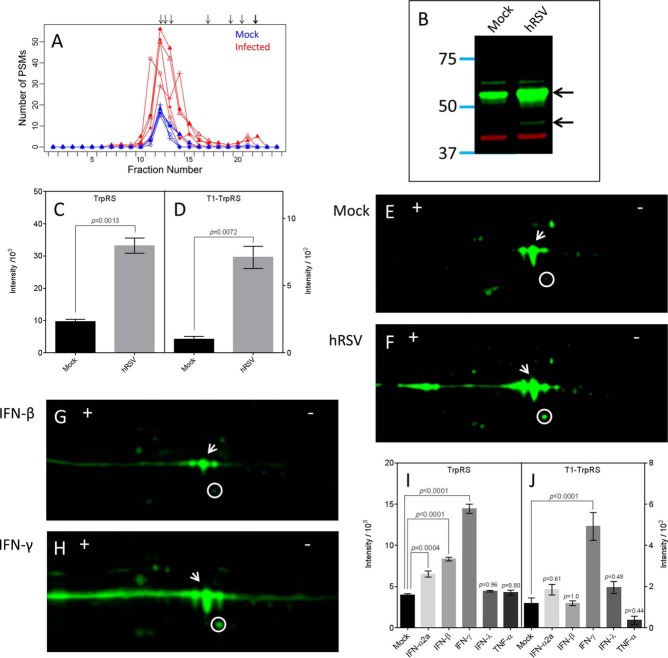
**Analysis of WARS proteoforms induced by hRSV infection and cytokine stimulation of A549 cells.** *A*, presents the observed focusing profile for the WARS protein group. Focusing for the five uninfected lysates and five hRSV-infected lysates are represented in blue and red, respectively. Arrows represent the predicted focusing for all proteoform sequences belonging to the WARS protein group. *B*, presents a representative image of one 1D-Western blot of analyses performed with four independent biological replicate sets of uninfected (Mock) and hRSV-infected (hRSV) A549 cell lysates. This image was obtained with equal quantities of protein from Mock and hRSV lysates from one replicate. The apparent full-length TrpRS and proteoforms are evident as green bands indicated by arrows with mobilities corresponding to 53.7 and 45.2 kDa, respectively. The red band represents β-actin used as a loading control. Quantitative data are presented in *C* and *D* for data obtained from the complete set of replicate 1D-Western blots of Mock (black bars) and hRSV (gray bars) lysates for the apparent full-length TrpRS proteoform (including the unresolved mini-TrpRS proteoform) and T1-TrpRS proteoform, respectively. Integrated intensities are presented as the mean ± S.E. (*n* = 4*).* Statistical analysis was performed using a paired, two-tailed Student's *t* test. *p* values are presented in both panels. Representative 2D-Western blots are shown for data obtained with equal quantities of *E* Mock, *F* hRSV, *G* IFN-β treated (IFN-β), and *H* IFN-γ treated (IFN-γ) lysates. Arrows and circles indicate the mobilities of the most abundant apparent full-length TrpRS and the apparent T1-TrpRS proteoforms, respectively. Quantitative data are presented in *I* and *J* for the apparent full-length TrpRS proteoform (including the unresolved mini-TrpRS proteoform) and T1-TrpRS proteoforms, respectively, from 1D-Western blots of replicates of cytokine treated A549 cells. Four independent replicate sets of treatments were analyzed. Integrated intensities are presented as the mean ± S.E. (*n* = 4). Statistical analysis was performed using a repeated measures one-way ANOVA with Dunnett's correction. *p* values compared with Mock are presented in both panels.

An increase in abundance and broadening of the focusing profile was apparent for the WARS protein group in the hRSV-infected lysates compared with the uninfected lysates (Fig. 7*A*). The WARS protein group had a group concordance of 0.95 for the uninfected lysates and 0.87 for the hRSV-infected lysates (Table I). Proteoform sequences with predicted focusing consistent with the observed PSMs were the full length Tryptophan-tRNA Synthetase (TrpRS) with the N-terminal methionine removed (P23381; 2–471), the cleaved form of TrpRS called T1-TrpRS (P23381; 71–471), and a splice variant called mini-TrpRS (P23381–2; 2–424). The presence of other proteoforms of WARS, namely T2-TrpRS (P23381; 94–471), T1-TrpRS (G3V4A3; 2–36), and T1-TrpRS (G3V4Q0; 2–40), were ruled out based on the very low concordance (Table I).

The relative contributions of full-length TrpRS, T1-TrpRS, and mini-TrpRS to the observed focusing profile was not resolved because of considerable overlap between the predicted focusing and substantial sequence identity. The combination of these three proteoform sequences however, was not sufficient to explain the broad IEF focusing profile for the hRSV-infected lysates as PSMs exclusively for the full-length TrpRS sequence spanned the majority of the observed focusing profile (supplementary Fig. S2*C*). Hence, Western blot technology was used to further resolve the WARS proteoforms.

Predominant WARS immunoreactivity was detected at ∼53.7 kDa by 1D-Western blotting of cell lysates (Fig. 7*B*) consistent with full-length TrpRS (55 kDa). A smear of immunoreactivity just below the main band could have represented mini-TrpRS which has a predicted size of ∼48 kDa. A discrete band of immunoreactivity was also apparent at 45.2 kDa in the hRSV-infected lysates, consistent with T1-TrpRS of 45.8 kDa. Quantitative analysis revealed that proteoforms of WARS in bands corresponding to full length TrpRS (plus mini-TrpRS) and T1-Trp-RS were induced upon infection (Figs. 7*C* and 7*D*).

The band corresponding to the full-length TrpRS at 53.7 kDa was detected as multiple spots on 2D-Western blots of both uninfected and hRSV-infected cells as was the smear corresponding to mini-TrpRS (Figs. 7*E* and 7*F*; white arrows). The multispot distribution of these species is likely to have been driven by PTMs. The number of and intensities of these immunoreactive spots was increased in hRSV-infected cells, which would account for the relative broadening of the focusing profile of the infected lysates observed by protein OGE. The induced T1-TrpRS proteoform was evident as a single spot in both uninfected (Fig. 7*E*; white circle) and hRSV-infected (Fig. 7*F*; white circle) cells, with enhanced relative intensity in the infected cells, indicating that it was unlikely to have been subjected to PTMs. Similar proteoform profiles were evident in 2D-Western blots of IFN-β (Fig. 7*G*) and IFN-γ (Fig. 7*H*) treated A549 cells, respectively. As observed previously (5, 26) the induction of full length TrpRS and T1-TrpRS by IFN-γ (Fig. 7*H*) was much more pronounced than by IFN-β (Fig. 7*G*). Mini-TrpRS also appeared to be induced by IFN-γ (Fig. 7*H*) but not IFN-β (Fig. 7*G*). Quantitative analysis of these various cytokine treatments by 1D-Western blotting confirmed the qualitative observations made from the 2D-Western blots (Figs. 7*I* and 7*J*). Of particular note was that the T1-TrpRS proteoform was significantly induced by IFN-γ but not by IFN-β or IFN-α2A (Fig. 7*J*). This cytokine induction profile was comparable to that observed with uninfected relative to hRSV-infected cells (Figs. 7*C* and 7*D*).

**Fig. 8.**
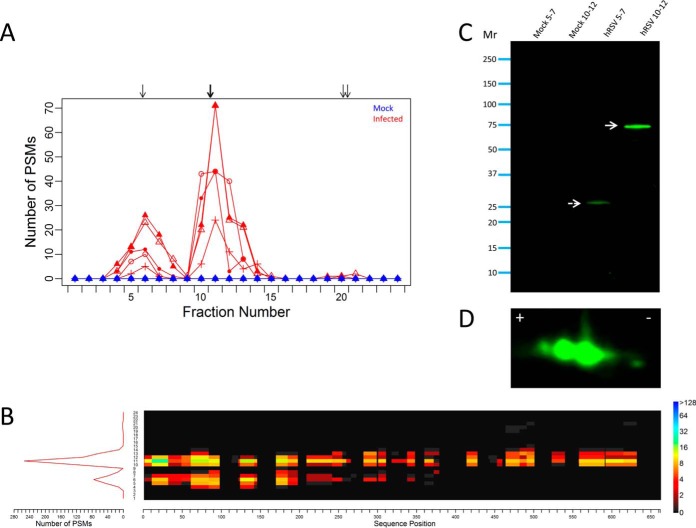
**Analysis of MX1 proteoforms induced by hRSV infection of A549 cells.** *A,* presents the observed focusing profile for the MX1 protein group. Focusing for the five uninfected and five hRSV-infected lysates are represented in blue and red, respectively. Arrows represent the predicted focusing for all proteoform sequences belonging to the MX1 protein group. *B*, depicts a sequence coverage heat map for the canonical MxA proteoform sequence (UniProt accession P20591) as presented by the sum of the five protein OGE separations of hRSV-infected A549 cells. The sequence coverage is plotted against the OGE fraction number using the color scale (presented to the right) to represent the total number of PSMs for the canonical MxA proteoform sequence. Focusing profiles representing the total number of PSMs matching MxA in the protein OGE fractionations of the infected cell lysates are plotted to the left of the heat map (red line). *C*, presents a 1D- Western blot of protein OGE fractions 5–7 and 10–12 of uninfected (Mock) and hRSV-infected (hRSV) A549 cells. The solid white arrow indicates the canonical full length proteoform of MxA of apparent *M*_r_ of ∼75 kDa and the broken white arrow indicates the truncated proteoform of MxA of apparent *M*_r_ of ∼29 kDa, as determined from the mobilities of the indicated molecular weight markers. *D*, presents the portion of a 2D- Western blot of an unfractionated hRSV-infected A549 cell lysate depicting the presence of immunoreactivity for the full length canonical proteoform of MxA in multiple spots of ∼75 kDa. The complete blot is presented as supplemental Fig. S6.

Two peaks of PSMs were observed for the MX1 protein group in hRSV-infected lysates. One peak centered at fraction six and the other centered at fraction eleven (Fig. 8*A*). No PSMs were observed for the MX1 protein group in uninfected lysates. The predicted focusing for the proteoform sequences belonging to the MX1 protein group was consistent with the two IEF peaks observed (group concordance of 0.86; Table I). The full length proteoform P20591 sequence (MxA) and an unreviewed splice variant F8W8T1 proteoform sequence were both predicted to focus to the IEF peak centered at fraction eleven. Only the full length MxA sequence explained all the PSMs observed in the predicted focusing window (fractions 10–12). However, the IEF peak centered at fraction eleven was broader than the predicted full length MxA focusing window (*i.e.* the IEF peak was greater than three OGE fractions wide). Consequently, PSMs for the MX1 protein group in fractions 13 and 14 were not explained but could be evidence of PTMs or an undefined MX1 proteoform.

The predicted focusing for a variant of MxA (varMxA; P20591–2) was sufficient to explain all of the PSMs observed in fractions five to seven. The varMxA proteoform is derived by alternative splicing of the MX1 mRNA. Consequently, the N-terminal 425 residues of the varMxA proteoform sequence match the N-terminal region of the full length P20591 MxA sequence exactly. From residue 426 the varMxA sequence diverges as a consequence of a frameshift and terminates as a 508 residue proteoform with a unique C terminus of 83 residues. No peptides matching the unique C-terminal 83 residues of varMxA were identified hence the variant sequence was not conclusively identified.

Two UniProt accession numbers corresponding to the MX2 gene (P20592 and B7Z5D3) matched three tryptic peptides belonging to the MX1 protein group (Table I). No peptides exclusively matching the MX2 proteoform sequences were identified (*i.e.* all peptides matching the MX2 proteoform sequences also matched MxA proteoform sequences). Both MX2 proteoform sequences were predicted to focus to fraction 20. Although a very small peak consisting of five PSMs was present in fractions 20 and 21 (Fig. 8*A*), none matched the MX2 proteoform sequences. Therefore, it is unlikely MX2 contributed to the complex focusing profile for the MX1 protein group.

The two IEF peaks apparent in the focusing profile for the MX1 protein group were consistent with the predicted focusing for at least two MxA proteoform sequences. The IEF peak centered at fraction 11 was consistent with the predicted focusing for the full length canonical MxA proteoform sequence in terms of pI and observed PSMs (Fig. 8*B* and Table I). Although the IEF peak centered at fraction six was consistent with the predicted focusing for varMxA, all the PSMs observed in this IEF peak also matched N-terminal peptides of the full length MxA proteoform sequence (Fig. 8*B*) and no PSMs were observed corresponding to the unique C-terminal portion of varMxA. Thus, the varMxA proteoform sequence was not unambiguously detected.

Western blotting was performed to further characterize the MxA proteoforms induced by hRSV infection using a polyclonal antiserum directed toward an epitope common to the N terminus of full length MxA and varMxA. MxA immunoreactivity was only observed in OGE fractions from hRSV-infected cell lysates. 1D-Western blots of protein OGE fractions ten to twelve revealed a prominent band of MxA immunoreactivity at ∼75 kDa (Fig. 8*C*) consistent with the essentially full-length MxA proteoforms. A band at ∼29 kDa was evident in fractions five to seven and no immunoreactivity corresponding to varMxA was observed at ∼55 kDa (Fig. 8*C*). The 29 kDa band would account for PSMs spanning up to approximately residue 260 of MxA (P20591), however, PSMs were observed spanning up to approximately residue 350 by direct analysis of the corresponding OGE fractions (Fig. 8*B*). These findings indicate that the 29 kDa proteoform is the predominant form of MxA in these fractions, however, the PSMs observed spanning residues 260 to 350 may provide evidence of a range of other less abundant truncated proteoform(s). Predicted focusing of *in silico* generated fragments of MxA indicated it is feasible that fragments produced via cleavages in the N-terminal region and at around residue 350 could focus to fractions five to seven (supplementary Fig. S4).

**Fig. 9.**
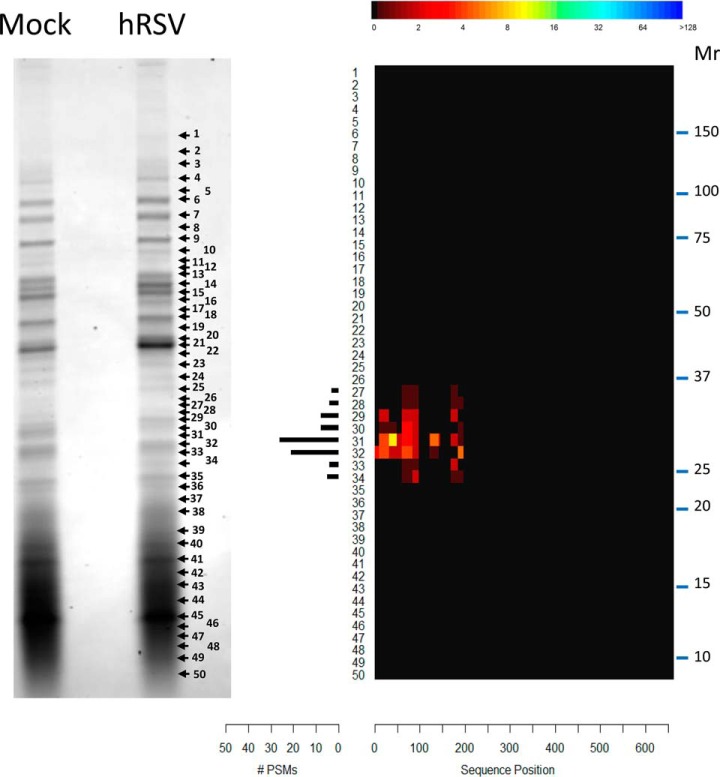
**Protomap analysis of MxA proteoforms in combined protein OGE fractions five to seven of hRSV-infected A549 cells.** Stained 1D-SDS-PAGE gels (left hand panel) of combined fractions five to seven from protein OGE of uninfected (Mock) and hRSV-infected (hRSV) A549 cell lysates were sliced according to the numbered arrows. Whereas in-gel digests of slices of the hRSV-infected fractions (right hand panel) revealed PSMs for MxA indicated in the PSM histogram, slices of the gel of the uninfected fractions produced no PSMs for MxA. The sequence coverage heat map (right) represents the alignment of all PSMs identified in the in-gel slices at each position of the canonical MxA proteoform sequence (P20591).

Protomap analysis of pooled protein OGE fractions five to seven corroborated the protein OGE results. MxA proteoforms in the size range of ∼25 kDa to 37 kDa were detected, with a predominant MxA proteoform of ∼29 kDa (Fig. 9). Notably, PSMs spanning from the N-terminal to residue 197 were observed. No evidence was detected to support the presence of varMxA.

One-dimensional Western blot and Protomap analysis of unfractionated whole cell lysates further corroborated the protein OGE observations. One dimensional Western blots revealed an intense band of immunoreactivity at ∼75 kDa consistent with the full length proteoform of MxA in whole cell lysates of infected but not the uninfected A549 cells (supplementary Fig. S5*A*). A smaller band of immunoreactivity of apparent *M*_r_ of 29 kDa was also observed uniquely in the infected cell lysates (supplementary Fig. S5*A*). No infection-specific MxA immunoreactivity was detected between 75 kDa and 29 kDa on infected or uninfected whole cell lysate blots (supplementary Fig. S5*A*). Protomap analysis of infected whole cell lysates revealed PSMs spanning the full length MxA proteoform sequence at 75 kDa and PSMs spanning a small portion of the MxA N terminus (residues 61–197) at ∼29 kDa (Fig. 5*C*). These Protomap data indicated that the predominant truncated proteoform of MxA in hRSV-infected A549 cells was a species of ∼29 kDa and that the truncated forms of MxA observed in protein OGE fractions five to seven were not an artifact of protracted sample processing steps.

The full-length form of MxA was detected in 2D-Western blots of hRSV-infected A549 whole cell lysates. This immunoreactivity was observed as two major spots as previously observed for MxA in hRSV-infected A549 cells (26) plus minor reactivity either side of these spots (Fig. 8*D*; supplementary Fig. S6). This multi-spot distribution probably accounts for the broadening of the protein OGE focusing profiles (Fig. 8*A*). No smaller proteoforms of MxA were detected on 2D-Western blots. No MxA immunoreactivity was detected in comparable blots of uninfected cell lysates.

Further characterization of MxA proteoforms present in hRSV-infected A549 cells was conducted by PCR analysis of transcripts of the MX1 gene. PCR was designed to amplify nucleotides 961 to 3426 based upon MX1 transcript variant 1 (RefSeq accession number NM_001144925.2), which would also include the complete coding sequences for UniProt accessions P20591, P20591–2, and F8W8T1 (supplementary Fig. S7). Whereas a product of 2465nt would have been produced by the full length MX1 transcripts a shorter product of 1980nt would have been expected for the transcript corresponding to varMxA. PCR analysis of MX1 transcription generated a single band of ∼2500 nucleotides following hRSV infection (supplementary Fig. S8). This indicated the presence of transcripts corresponding to the full length coding sequence of MX1 only and no evidence of varMxA or other transcripts from the MX1 gene. Sequence analysis of the PCR product (supplementary Fig. S9) translated *in silico* into the full length MxA protein sequence (supplementary Fig. S10). Overall, these data indicate that MxA exists in hRSV-infected A549 cells mainly as the full length 75 kDa proteoform with multiple PTMs plus a truncated proteoform of ∼29 kDa and potentially less abundant truncated proteoform(s) of up to 37 kDa.

##### Proteoform Regulation Within Protein Groups Not Regulated on a Global Level by hRSV Infection

Regulation of proteoforms following hRSV infection may not be detected as a global difference in abundance, however, regulation by other means, such as PTMs, may result in proteoform redistribution. To identify protein groups where the observed focusing profiles were different in uninfected compared with hRSV-infected A549 cells, the KSb test was applied. According to the KSb test, the HSPB1 protein group ranked highly as a protein group regulated by proteoform redistribution (supplementary Table S2).

**Fig. 10.**
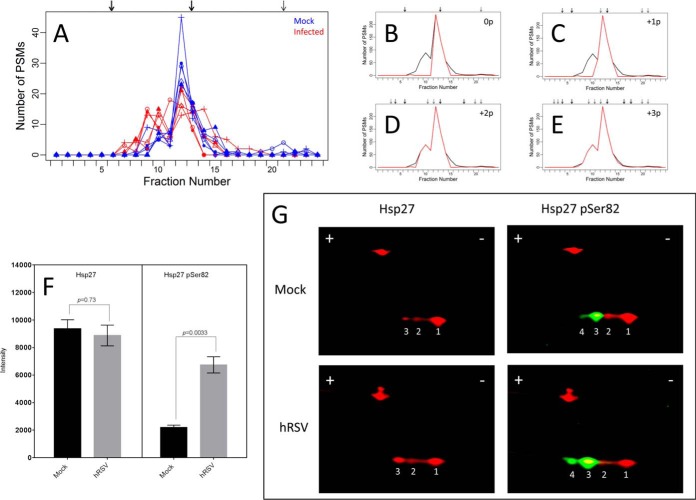
**Analysis of Hsp27 proteoforms induced by hRSV infection of A549 cells.** *A*, presents the observed focusing profiles for the HSPB1 protein group. Focusing for the five uninfected (Mock) and five hRSV-infected (hRSV) lysates are represented in blue and red, respectively. Arrows represent the predicted focusing for all proteoform sequences belonging to the HSPB1 protein group. *B–E*, present the observed focusing profile for the protein group as a black line and is equal to the number of PSMs for the protein group in each OGE fraction, where the sum is taken over the ten protein OGE separations of Mock and hRSV lysates. The red lines represent the portion of PSMs that can be explained by the concordance analysis allowing for no modification (concordance of 0.6), up to one phosphorylation (concordance of 0.7), up to two phosphorylations (concordance of 0.91), and up to three phosphorylations (concordance of 0.96), respectively. *F*, depicts quantitative data obtained from the complete sample set of Mock (black bars) and hRSV (gray bars) lysates. This sample set was independent to that used to produce data presented in *A*. Generic Hsp27 and Hsp27 pSer82-specific reactivities are presented in the left hand and right hand panels, respectively. Integrated intensities are presented as the mean ± S.E. (*n* = 4*).* Statistical analysis was performed using a paired, two-tailed Student's *t* test. *p* values are presented in both panels. *G*, presents 2D-Western blots of equal quantities of protein from lysates of one replicate of Mock (top panels) and hRSV (bottom panels) A549 cells used to produce the quantitative blotting data presented in *F*. General Hsp27 reactivity was evident as red spots 1–3 (left hand panels) and Hsp27 pSer82 reactivity was evident as green spots 3 and 4 (right hand panels) when the blots from the left hand panels were reprobed with the Hsp27 pSer82-specific antibody. The red spots to the top left of each panel with mobilities corresponding to ∼42 kDa represents β-actin used as a loading control.

The predicted focusing for proteoform sequences belonging to the HSPB1 protein group were sufficient to explain a greater proportion of the observed focusing profile for the uninfected lysates compared with the hRSV-infected lysates (group concordance of 0.72 compared with 0.49; Table I). The HSPB1 protein group consists of three proteoform sequences (Figs. 10*A* and 10*B*). The Hsp27 proteoform sequence (P04792) matched all PSMs belonging to the HSPB1 protein group and was sufficient to explain the observed IEF peak at fraction 12. The remaining two proteoform sequences F8WE04 and C9J3N8 are unlikely to have contributed to the observed focusing profile, because no PSMs matched the proteoform sequences exclusively and concordance was less than 0.01 for these proteoforms (Table I). Allowing phosphorylated proteoforms to contribute to the observed focusing profile, the concordance for the HSPB1 protein group increased from 0.6 to 0.7, 0.91 and 0.96 by allowing for up to one, up to two and up to three phosphorylations, respectively (Figs. 10*B*–10*E* and Table I).

Quantitative 1D-Western blotting of lysates of uninfected and hRSV-infected A549 cells using a generally reactive antibody to Hsp27 (UniProt accession P04792) confirmed that Hsp27 is not regulated at the global protein level by hRSV infection (Fig. 10*F*). However, blotting indicated that hRSV infection of A549 cells induced phosphorylation of Ser82 (Fig. 10*F*). Probing of 2D-Western blots of uninfected and infected cell lysates with the generally reactive Hsp27 antibody revealed a major spot of reactivity (Fig. 10*G*, spot 1 in the left hand panels) and two less intense spots (Fig. 10*G*, spots 2 and 3 in the left hand panels) that were more acidic than the major spot. Spot three produced pronounced reactivity with the Hsp27 pSer82-specific antibody (Fig. 10*G*, right hand panels). Weaker Hsp27 pSer82 reactivity was evident for a fourth spot (Fig. 10*G*, spot 4 on right hand panels). Spots 2 and 3 of the infected samples exhibited increased relative intensities with the generally reactive Hsp27 antibody compared with the uninfected samples (Fig. 10*G*, left hand panels). The Hsp27 pSer82-specific antibody revealed more pronounced reactivities for spots 3 and 4 of the infected compared with the uninfected samples (Fig. 10*G*, right hand panel).

## DISCUSSION

Recent advances for in-depth characterization of cellular proteomes have been driven by new mass spectrometry platforms (5, 6, 26) and bioinformatic tools (10, 36–38, 46–48). It is now a realistic expectation that greater than 5000 protein groups can be identified and quantified in a single bottom-up MS/MS analysis of submicrogram quantities of unfractionated whole cell lysates (6). This has had an enormous influence on our ability to define the proteomic compositions and dynamics of cellular systems on a global level (5, 6, 49). However, an even deeper understanding would be forthcoming if cellular proteomes could be more readily defined at the proteoform level (4).

Computational strategies have been described for discovering proteoforms in bottom-up proteomic data sets (14, 15) but these have potential limitations. Top-down proteomics involving HPLC-MS/MS of intact proteins has the potential to characterize proteoforms (4, 19, 20), however it is not yet as universally attainable in most laboratories as bottom-up approaches.

Proteoform profiling has been achieved by intact protein separation followed by bottom-up MS/MS analysis (7, 26, 50). Differential 2D-gel separation has the potential to identify differentially migrating proteoforms and detect differences between cellular proteomes at the proteoform level (26, 50). This approach takes advantage of high resolution IEF in the first dimension followed by orthogonal SDS-PAGE. However, 2D-gel approaches require complex in-gel sample processing steps to produce samples for bottom-up MS/MS analyses. One-dimensional protein separation may also be used for proteoform identification. For example, Protomap analysis utilizes 1D-SDS-PAGE to differentiate between proteoforms based on molecular size (22, 23). Protomap also requires complex in-gel processing steps.

The proteoform profiling strategy described herein is based on 1D-intact protein separation by protein OGE followed by bottom-up HPLC-MS/MS analysis of individual OGE fractions and qualitative assessment of the resultant distribution of protein groups. As distinct from molecular size-based fractionation (22, 23), which requires significant molecular size differences to distinguish between proteoforms, the present proteoform profiling strategy exploited the possibility that a more subtle distinction between proteoforms can be achieved by virtue of pI differences.

The data set used to assess the proposed strategy included whole cell lysates of five matched sets of uninfected and hRSV-infected A549 cells that were individually separated into 24 fractions. Each fraction was digested with trypsin and analyzed by bottom-up HPLC-MS/MS (5, 26). Efficient and reproducible focusing by protein OGE was evident from the observation that ∼67% of the focusing profiles analyzed matched narrow focusing templates. Furthermore, experimentally observed migration of proteoforms was consistent with the 3–11 nonlinear reference pH gradient used for the separations. Consequently, it was possible to develop the strategy described herein to profile the proteoform compositions of complex protein mixtures based on observed focusing profiles in addition to concordance and statistical analyses.

The advantage of the proteoform profiling approach reported herein over 2D-DIGE is directly exemplified by comparison with an independent 2D-DIGE analysis of hRSV infected A549 cells (26). It is noteworthy that 3412 protein groups were identified across the ten protein OGE separations used herein for proteoform profiling, of which 114 were previously identified as differentially regulated at the total sample level (FDR < 1%). Proteoform profiling of this data set described herein indicates that this corresponds to hRSV regulation of greater than 114 proteoforms (Fig. 3 and supplementary Table S2). This compares to the detection of only 1681 discrete spots defined by 2D-DIGE (26) of which only 58 spots were differentially regulated in the comparison of uninfected to wild-type hRSV-infected cells (α<0.01 and a fold change of at least 1.2). Of the 58 regulated spots, only 13 yielded identifications of interest (the remaining spots were not identified or were bovine contaminants). The resolution of 2D-DIGE was superior to protein OGE protein separation, as exemplified by multiple full-length MxA proteoforms resolved by 2D-DIGE (26) compared with a broad peak spanning fractions 10–14 by protein OGE (Fig. 8*A*). The pH difference between adjacent OGE fractions was ∼0.11–0.58 pH units (supplementary Fig. S1*A*), indicating that the resolution of the OGE separation was ∼0.2 pH units in the flattest region of the pH gradient. Despite this, the protein OGE-derived proteoform profiles indicated proteoform diversity for full-length MxA, which was confirmed using 2D-Western blot analysis (Fig. 8*D*). Furthermore, the protein OGE resolution could potentially be improved by increasing the number of well and/or using narrower range immobilized pH gradients (30).

Although protein OGE separation has lower resolution than the 2D-gel approach, it provided a better assessment of proteome regulation on both global (5) and proteoform perspectives. The superior performance of the protein OGE approach could relate to several factors. For example, 2D-gel approaches detect regulation of discrete spots then determines spot compositions using a complex series of inefficient in-gel processing steps. By comparison, data are collected on all proteins using more straight-forward in-solution processing steps with the protein OGE approach and regulation is identified post data acquisition. This may explain the greater protein sequence coverage of MxA proteoforms achieved in the present study after protein OGE separation (Fig. 8*B*) compared with Protomap (Fig. 9 and supplementary Fig. S5*C*) in the present study or by 2D-DIGE previously (26). Comigration of proteoforms can result in sampling bias with 2D-gel approaches (26, 50). This could result in concealment of regulation or interference of spot composition determination. By comparison, the protein OGE based approach described herein allows unbiased sampling of comigrating proteoforms. Finally, proteins may simply escape detection in the 2D-gel approach because of low abundance or poor staining (50, 51).

Complementary analytical methods may be required to fully define proteoform diversity discovered by proteoform profiling. Unbiased approaches such as Protomap (as in Fig. 9), or targeted approaches using a combination of protein, proteoform and/or PTM-specific antibodies for 1D- and 2D-Western blotting (as in Figs. 8*C*, 8*D*, 10*G*) may be required to deconvolute proteoform diversity and localize PTM sites. A significant advantage of protein OGE compared with 2D-gel approaches is that MS data acquisition consumes only a small portion of the protein OGE fractions. Consequently, sufficient residual quantities of each fraction are available for multiple MS data acquisitions and orthogonal analyses to address proteoform specific questions, as demonstrated herein (Figs. 8*C*, 8*D*, 9 and 10*G*). Other strategies such as top-down analyses can also take advantage of protein OGE prefractionation, by reducing the deleterious effects of sample complexity on ionization and data acquisition.

Within the present study, several protein groups that were comprised of only one proteoform sequence such as ZNF259, IFIT3, SERPINB9, and IFIT2 (Figs. 4*A*, 4*C*, 4*D*, and 4*G*, respectively) exhibited narrow in-solution focusing in accordance with the calculated pIs. For some protein groups with multiple proteoform sequences, such as TUBG1, IDI1, and ISG20 (Figs. 4*E*, 4*F*, and 4*H*, respectively), it was possible to establish which proteoform sequences were likely to explain the observed PSMs. Furthermore, for some protein groups, precursor and processed proteoforms were distinguishable. For example, the observed focusing profile for the NENF protein group (Fig. 4*B*) matched the predicted focusing for the proteoform sequence with the signal peptide removed but not the unprocessed proteoform.

Other protein groups required the presence of more than one proteoform to explain the observed focusing profiles which in general had broadened and/or multiple peaks (*e.g.* IKBIP, SEPT9, PCMT1, CALU, CTSL1, PSMB10, STAT1, WARS, MX1, and HSPB1). Concordance analyses demonstrated that some proteoform sequences were unlikely contributors to the observed focusing profiles and, hence, were likely to be of very low abundance or absent from the lysates. For the PSMB10 protein group there was evidence supporting the presence of both the precursor and the proteoform sequence with the propeptide removed (Fig. 5*E*; supplementary Fig. S2*A,* S3). These PSMB10 proteoforms were validated using orthogonal methods (supplementary Fig. S3) such as 1D- and/or 2D-Western blotting. In other cases, such as STAT1, WARS, MX1, and HSPB1 protein groups, the focusing profiles were very complex and these orthogonal methods greatly aided deconvolution of the apparent proteoform diversity.

Importantly, it was possible to gain insights into hRSV infection-specific regulation of proteoforms using the proteoform profiling strategy described above. Evidence of hRSV regulation of only one proteoform was evident for IFN-inducible protein groups such as SERPINB9, IFIT2 and IFIT3. Insight was obtained for protein groups that exhibited broad focusing or focused into multiple peaks consistent with hRSV induction and/or PTM of multiple proteoforms. Protein groups with evidence of hRSV-induction of multiple proteoforms included PSMB10, CTSL1, STAT1, WARS, MX1, and HSPB1. It was evident that the proteoform profiling strategy described herein was more effective at identifying and defining virus-specific proteoform regulation than was possible using 2D-DIGE approach (26). In particular, no proteoforms of PSMB10, CTSL1, STAT1, WARS, MX1, or HSPB1 were seen to be differentially regulated in wild-type hRSV-infected A549 cells compared with uninfected cells using the 2D-DIGE approach (26). Interestingly, in A549 cells infected with nonstructural protein1 gene deficient hRSV, both WARS and MxA were distributed into two up-regulated spots (26). However, these spots corresponded to the sizes of the unprocessed proteoforms and the smaller processed proteoforms observed in the present study were not detected on the 2D-gels (26).

STAT1 is a key transcription factor for type I, II, and III IFN signaling (52, 53). STAT1 participates in type I and III IFN signaling as a heterodimer with STAT2 (52, 53) and in type II IFN (IFN-γ) signaling as a homodimer (53). Stat1-α and Stat1-β proteoforms result from alternative splicing which deletes a portion of the C-terminal transactivation domain of Stat1-α to produce the shorter Stat1-β proteoform (52, 54). Recent studies have shown that Stat1-β is involved in type II IFN signaling (52, 54). Thus, the observed induction of Stat1-β in the present study is consistent with hRSV induction of IFN-γ responses in A549 cells (5, 26). The STAT1 example serves to exemplify how the proteoform profiling approach described herein can highlight profiles that are indicative of regulation by alternate splicing and PTMs.

The major proteoform of WARS evident in A549 and other cells was the full length TrpRS of 55 kDa (55, 56). The present data show that in addition to full length TrpRS, WARS proteoforms exist in A549 cells because of alternative splicing (*e.g.* a species of ∼48 kDa known as mini-TrpRS (55, 57)), proteolysis (*e.g.* T1-TrpRS of ∼47 kDa (57)), and PTMs (58). Consistent with the present findings, full length TrpRS and min-TrpRS have previously been shown to be induced more by IFN-γ than by type I and III IFNs (5, 26, 55, 56, 59). It is also evident from the work herein that the proteolytically produced T1-TrpRS proteoform is also induced in A549 cells treated with IFN-γ but not type I or III IFNs. The pronounced induction of full length TrpRS, mini-TrpRS and particularly T1-TrpRS in hRSV-infected A549 cells supports the previous conclusions that hRSV induces an IFN-γ response in A549 cells (5, 26, 60). The induction of different WARS proteoforms in hRSV-infected cells may be a result of Stat1-β induction described above. Thus proteoform profiling highlighted regulation of WARS proteoforms that were found by downstream targeted Western blotting analysis to involve a complexity of abundance changes, alternate splicing and PTMs, including proteolysis. The canonical function of TrpRS, mini-TrpRS, and T1-TrpRS is to catalyze the charging of tryptophanyl-tRNAs with tryptophan, which would support both cellular and viral protein biosynthesis (61). However, WARS has secondary roles including regulation of transcription and translation. Mini-TrpRS and proteolytically derived proteoforms T1-TrpRS and T2-TrpRS are also secreted from cells and exert extracellular angiostatic cytokine activity (57). It is plausible that such cytokine activity may contribute to the pathophysiology of hRSV infection.

The canonical MxA proteoform of ∼75–76 kDa has structural and functional similarities to dynamin (62, 63) and is regarded as the most potent antiviral protein known (62, 63). The shorter proviral varMxA proteoform has been shown to be produced by alternate splicing in human fibroblasts infected with the DNA virus, HSV-1 (64). The full length 75 kDa proteoform MxA was definitively identified in fractions ten to 12 of hRSV-infected A549 cell lysates, albeit with a broadened focusing profile, apparently because of PTMs. A range of smaller proteoforms was observed in fractions five to seven with predominance of a species at ∼29 kDa. The splice variant varMxA was predicted to focus to fraction six, however, no evidence was observed at the corresponding size of 55 kDa. Furthermore, the presence of MxA splice variants, including varMxA, was ruled out based on PCR performed on hRSV-infected A549 cells.

Peptides spanning the first 350 residues of the full length sequence were evident in protein OGE fractions five to seven. Cleavage of MxA following residue 350 would result in a proteoform of MxA of ∼38 kDa that was predicted to focus to fraction five (supplementary Fig. S4). However, Western blotting of protein OGE fractions five to seven detected a predominant proteoform of 29 kDa. Futhermore, Protomap analysis of unfractionated infected lysates also revealed MxA proteoforms of 75 kDa and 29 kDa. Therefore, it appears likely that the predominant truncated proteoform of MxA produced in hRSV-infected A549 cells was a consequence of proteolysis of the full length proteoform at around residue 260. However, it was not possible to rule out the presence of less abundant truncated proteoforms involving alternate cleavage(s). *In silico* digestion of MxA revealed that fragments containing the N-terminal region of MxA are predicted to focus to fractions four to nine (supplementary Fig. S4). Furthermore, fragments predicted to focus to fractions five to seven predominantly span no further than residue 440. Short fragments that do not contain the MxA N-terminal region are predicted to focus beyond fraction ten (*e.g.* the fragment spanning ∼260 to 350 is predicted to focus to fraction 15).

Monomeric MxA units form tetramers which associate further to form higher order supramolecular rings which encompass viral nucleocapsids to exert antiviral activity by direct sequestration and/or exerting mechanical disruption (62, 63). Truncation of MxA by proteolysis would be expected to deplete hRSV-infected cells of this potent antiviral protein and partially explain the suppression of antiviral responses to hRSV infection. MxA proteoform characterization represents an example of protoform discovery that would not have been achieved via recently described computational analyses of global bottom-up proteomic data sets (14, 15).

The finding of hRSV-induced redistribution of HSPB1 proteoforms exemplifies another advantage of the proteoform profiling strategy described herein. Hsp27 was not seen to be regulated by hRSV infection on global or proteoform levels in previous studies (5, 26). However, phosphorylation of Hsp27 at serines 78 and 82 has previously been correlated with hRSV induced increased epithelial cell membrane permeability and the pathophysiology of hRSV (65). The unbiased profiling strategy described herein suggested regulation of Hsp27 proteoforms by PTMs such as phosphorylation. Phosphorylation of at least one of the previously described phosphorylation sites on Hsp27, namely Ser82, was confirmed by 2D-Western blotting. Detection of Hsp27 proteoform regulation exemplifies the utility of KSb statistical analyses for the identification of hRSV-induced proteoform redistribution.

In summary, the present study demonstrated that protein OGE of whole cell lysates achieves highly effective and reproducible protein separation. Furthermore, bottom up MS analysis and fraction by fraction computational analyses can facilitate informative proteoform profiling. Using the proteoform profiling strategy described herein, it is possible to discover and identify proteoform sequences present in a complex proteome. Furthermore, it is also possible to identify focusing profiles that reflect proteoform regulation because of PTMs, including proteolysis and phosphorylation, that may be of functional significance. Western blotting technologies and Protomap analysis were used for validation and deconvolution where required within the present profiling strategy. However, it is feasible that other proteoform profiling approaches (14, 15, 22, 23), SRM (18) and/or top-down techniques could also be used to complement the present approach. The proteoform profiling strategy described herein may be of generic utility for gaining a better understanding of the interactions of a broad range of viruses with their host cells and be generally applicable to the analysis of other biological systems. Thus, this proteoform profiling strategy could be used to select targets for downstream research efforts involving a range of biological questions.

## Supplementary Material

Supplemental Data

## Footnotes

^1^ The abbreviations used are:

PTM: post-translational modification
1D: one-dimensional
2D: two-dimensional
HSPB1: heat shock protein beta-1 protein group
Hsp27: heat shock protein 27
Hsp27 pSer82: PhosphoSerine 82 form of Hsp27
hRSV: human respiratory syncytial virus
IFN: interferon
MOI: multiplicity of infection
KSb: bootstrapped two-sample Kolmogorov-Smirnov test
MS: mass spectrometry
MS/MS: tandem mass spectrometry
OGE: Off-Gel Electrophoresis
pfu: plaque forming units
PSM: peptide-to-spectrum match
STAT1: Signal transducer and activator of transcription1 protein group
Stat1-α: Alpha proteoform of Signal transducer and activator of transcription1
Stat1-β: Beta proteoform of Signal transducer and activator of transcription1
UniProt: Universal Protein Resource
UniProtKB: Universal Protein Resource Knowledgebase
WARS: Tryptophan-tRNA synthetase protein group
TrpRS: Tryptophan-tRNA synthetase proteoform
Mini-TrpRS: alternate splice proteoform of Tryptophan-tRNA synthetase
T1-TrpRS: T1 proteoform of Tryptophan-tRNA synthetase
T2-TrpRS: T2 proteoform of Tryptophan-tRNA synthetase.
