# Supplementary material for: Proteoform-Specific Insights into Cellular Proteome Regulation
Source: Mol Cell Proteomics. 2016 Jul 22;15(10):3297–320. doi: 10.1074/mcp.O116.058438 (PMC5054351; doi:10.1074/mcp.O116.058438)
Supplement: Supplemental Data [file supp_15_10_3297__index.html]

Proteoform-specific insights into cellular proteome regulation — Proteoform-Specific Insights into Cellular Proteome Regulation — Profiling of Cellular Proteoforms — Supplemental Data 

# Proteoform-Specific Insights into Cellular Proteome Regulation

## Supplemental Data

- Supplemental Table 1 (.pdf, 27 KB) - The number of pI values represented in the boxplots presented in Supplemental Figure 1B for each fraction for the ten protein OGE separations.
- Sup. Fig. (.pdf, 1.4 MB) - Supplemental Figures
