# Supplementary material for: Proteoform-Specific Insights into Cellular Proteome Regulation
Source: Mol Cell Proteomics. 2016 Jul 22;15(10):3297–320. doi: 10.1074/mcp.O116.058438 (PMC5054351; doi:10.1074/mcp.O116.058438)
Supplement: Supplemental Data [file 10.1074_O116.058438_mcp.O116.058438-1.pdf]

Supplemental Table 1. The number of pI values represented in the boxplots presented in Supplemental Figure 1B for each fraction for the ten protein OGE separations.

IEF Fraction Number	Sample									
	hRSV 1	hRSV 2	hRSV 3	hRSV 4	hRSV 5	Mock 1	Mock 2	Mock 3	Mock 4	Mock 5
F1	0	0	6	0	0	0	0	0	0	0
F2	1	0	1	4	2	2	0	1	0	0
F3	15	12	16	15	16	21	5	13	9	8
F4	27	18	33	25	32	32	21	39	32	28
F5	30	33	47	29	32	44	26	44	26	17
F6	26	35	31	23	27	31	32	36	32	34
F7	25	31	33	29	31	31	28	30	27	14
F8	25	32	32	46	46	29	31	31	34	44
F9	34	35	33	41	12	29	32	30	35	36
F10	33	38	41	46	51	35	35	42	42	47
F11	16	36	24	29	38	27	35	32	35	34
F12	7	9	42	26	21	39	38	38	37	40
F13	5	46	23	45	54	37	40	43	37	29
F14	20	35	28	26	30	13	34	35	32	31
F15	4	43	28	42	42	39	54	31	37	42
F16	27	50	32	24	19	21	41	36	34	37
F17	4	37	27	16	19	17	15	29	38	36
F18	13	6	18	29	29	23	19	36	20	19
F19	0	2	18	30	33	26	25	2	39	26
F20	10	1	21	45	47	16	19	3	37	43
F21	5	34	7	41	25	3	9	7	19	29
F22	22	9	0	2	21	3	1	15	13	27
F23	7	0	0	0	0	0	0	0	17	11
F24	0	0	1	0	0	0	0	0	1	1
