# Supplementary material for: Proteoform-Specific Insights into Cellular Proteome Regulation
Source: Mol Cell Proteomics. 2016 Jul 22;15(10):3297–320. doi: 10.1074/mcp.O116.058438 (PMC5054351; doi:10.1074/mcp.O116.058438)
Supplement: Supplemental Data [file 10.1074_O116.058438_mcp.O116.058438-2.pdf]

Supplemental Fig. 1. **Agreement between the experimental pI values and the reference pH gradient.**

Five hRSV-infected lysates (hRSV) and five mock-infected lysates (Mock) were individually separated into 24 fractions using protein OGE. For each OGE separation, the calculated pI values for selected proteoform sequences observed to focus to each OGE fraction were used to represent the experimental pI values.

In the following gradient plots, the red line represents the 3-11 nonlinear reference pH gradient (Immobiline DryStrip pH 3-11 non-linear gradient, GE Healthcare). To represent the experimental fractionation patterns, the distribution of pI values for each OGE fraction is superimposed. A boxplot is used to represent the distribution of pI values for each fraction, with the exception of fractions that had less than ten pI values, in which case all pI values were plotted. The number of pI values representing each OGE fraction is presented in Supplemental Table 1.

The boxplots were generated using the "boxplot" function from the graphics package in R (version 2.15.1 (1)). Accordingly, the median pI is represented as a thick black line that is surrounded by a box that represents the interquartile range (i.e., the median ± 25% of the data). The whiskers represent the range of pI values observed in the fraction and open circles represent outliers. Here an outlier is defined as a pI value that is greater than 1.5 times the interquartile range above the upper quartile or below the lower quartile.

Reference:

1. R Core Team (2016). R: A language and environment for statistical computing. R Foundation for Statistical Computing, Vienna, Austria.

A) Reference pH gradient

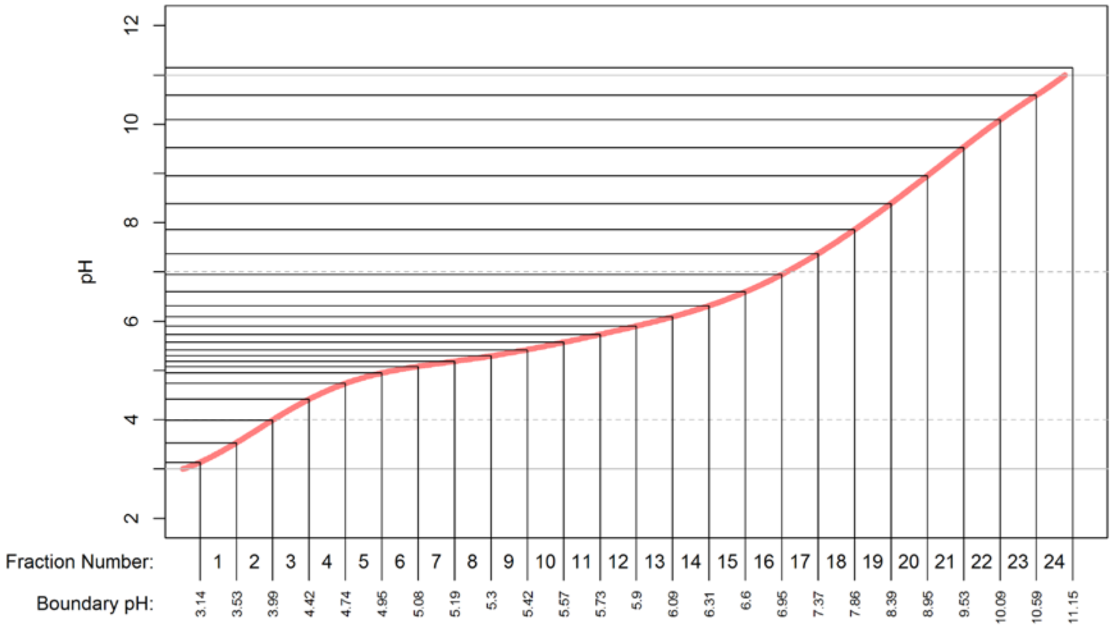

B) Agreement between the distribution of experimental pI values and the reference pH gradient for the ten OGE separations. Five replicate sets of uninfected (Mock) and hRSV-infected A549 cell lysates were fractionated.

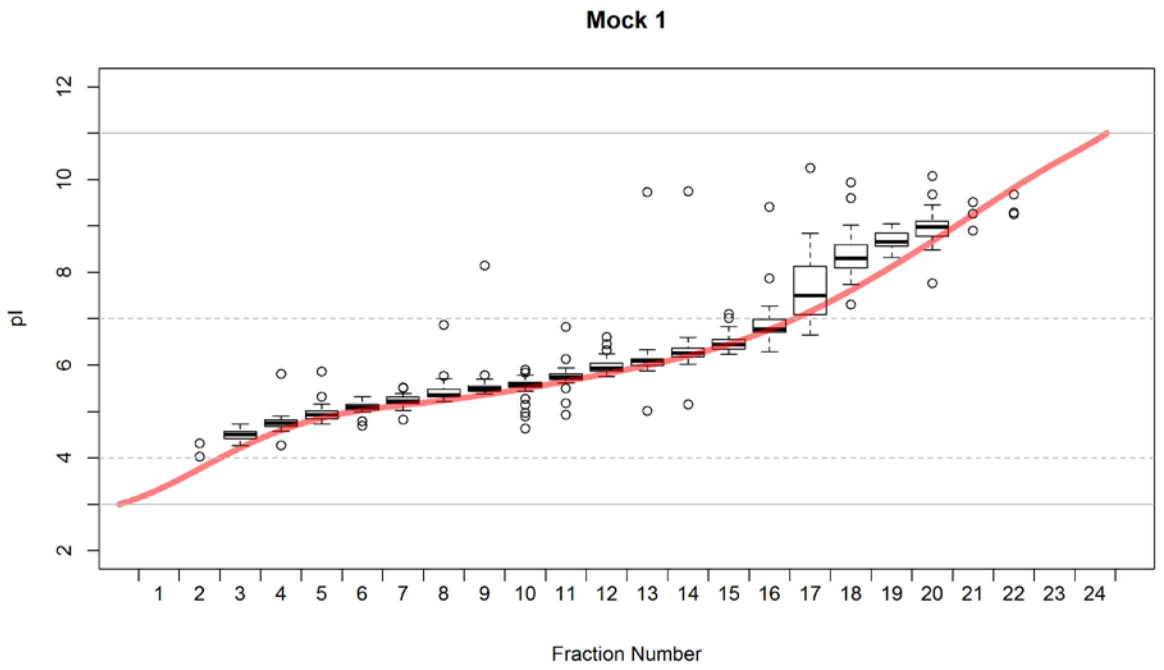

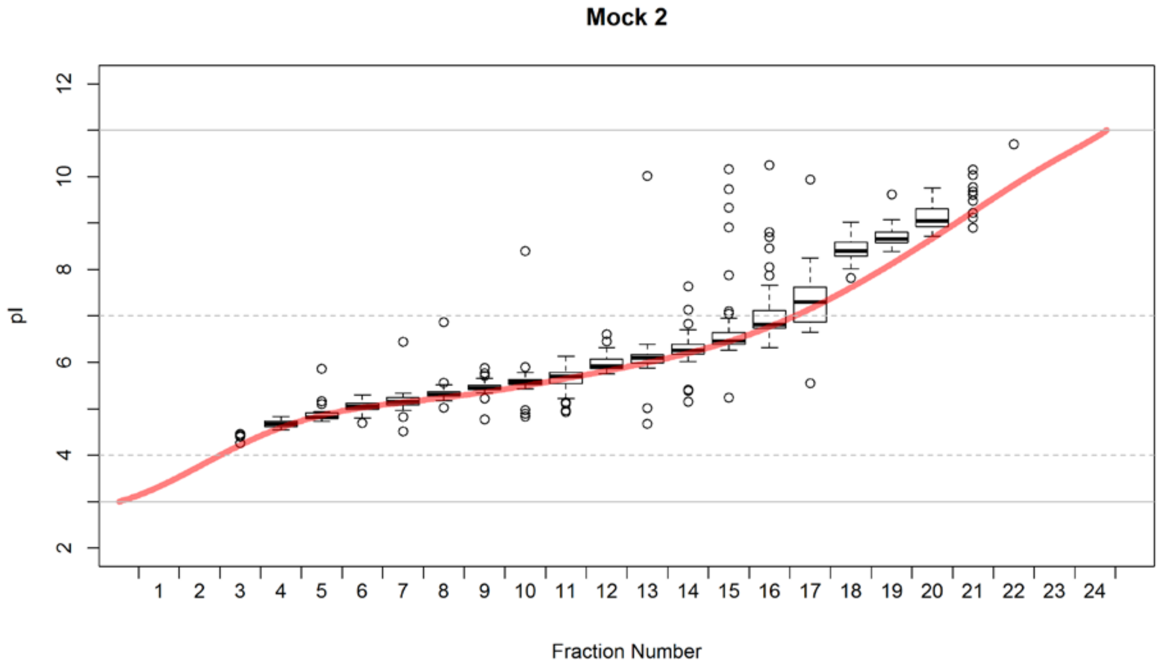

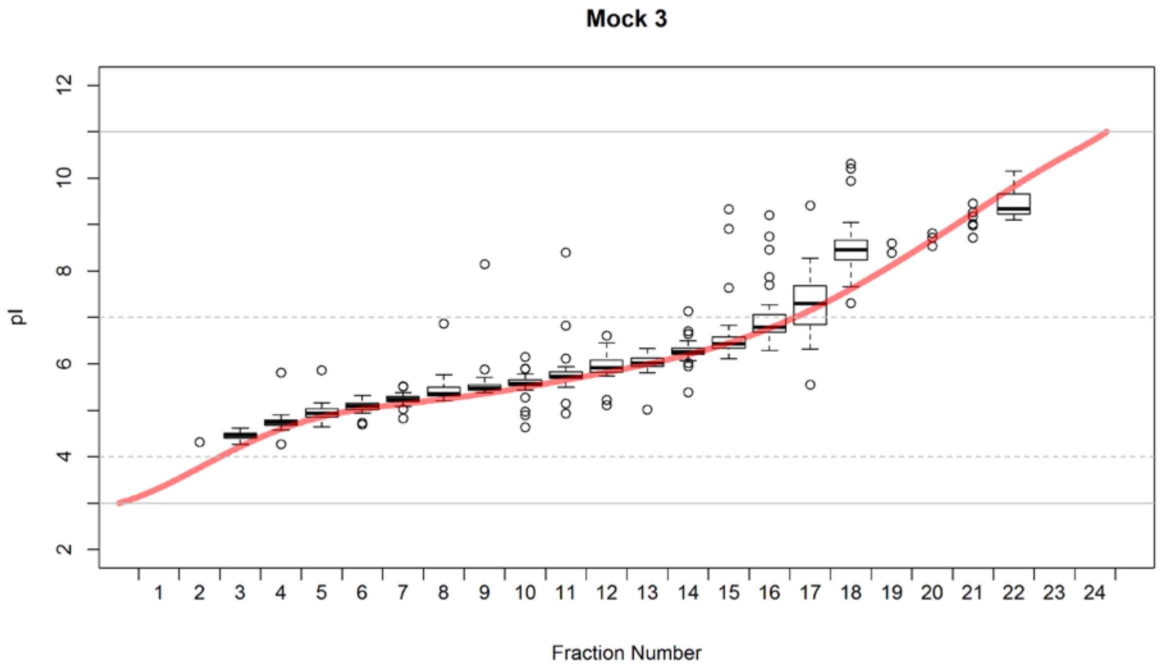

Mock 3
pI
2
4
6
8
10
12
1 2 3 4 5 6 7 8 9 10 11 12 13 14 15 16 17 18 19 20 21 22 23 24
Fraction Number

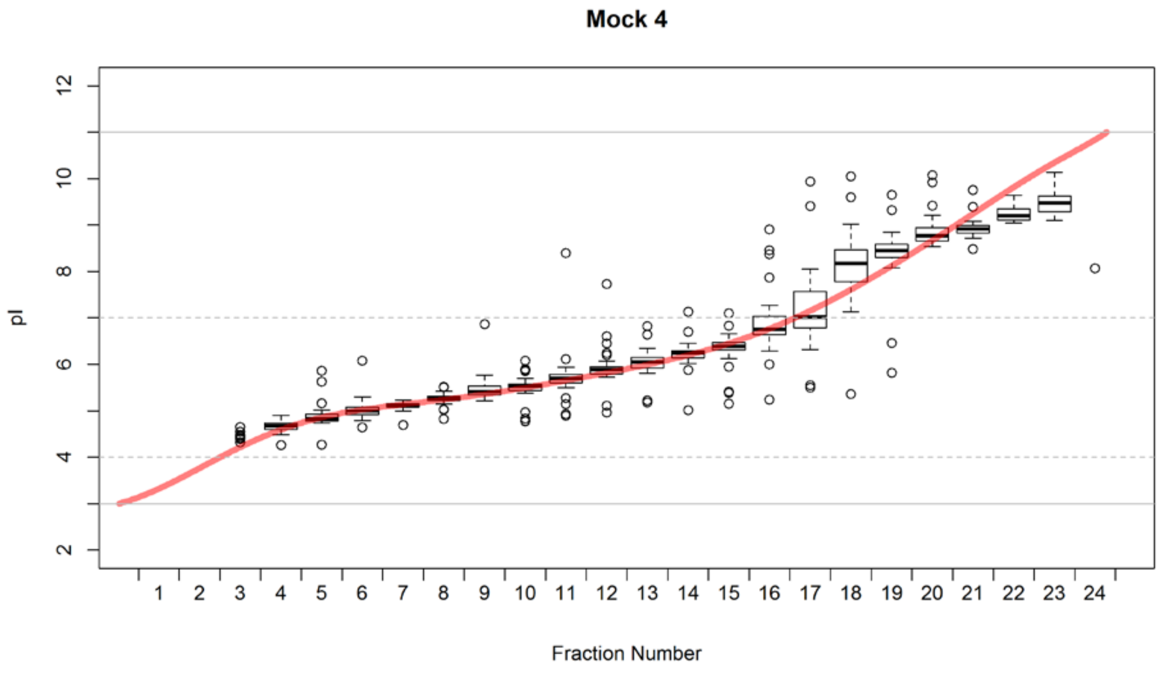

Mock 4
pI
2
4
6
8
10
12
1 2 3 4 5 6 7 8 9 10 11 12 13 14 15 16 17 18 19 20 21 22 23 24
Fraction Number

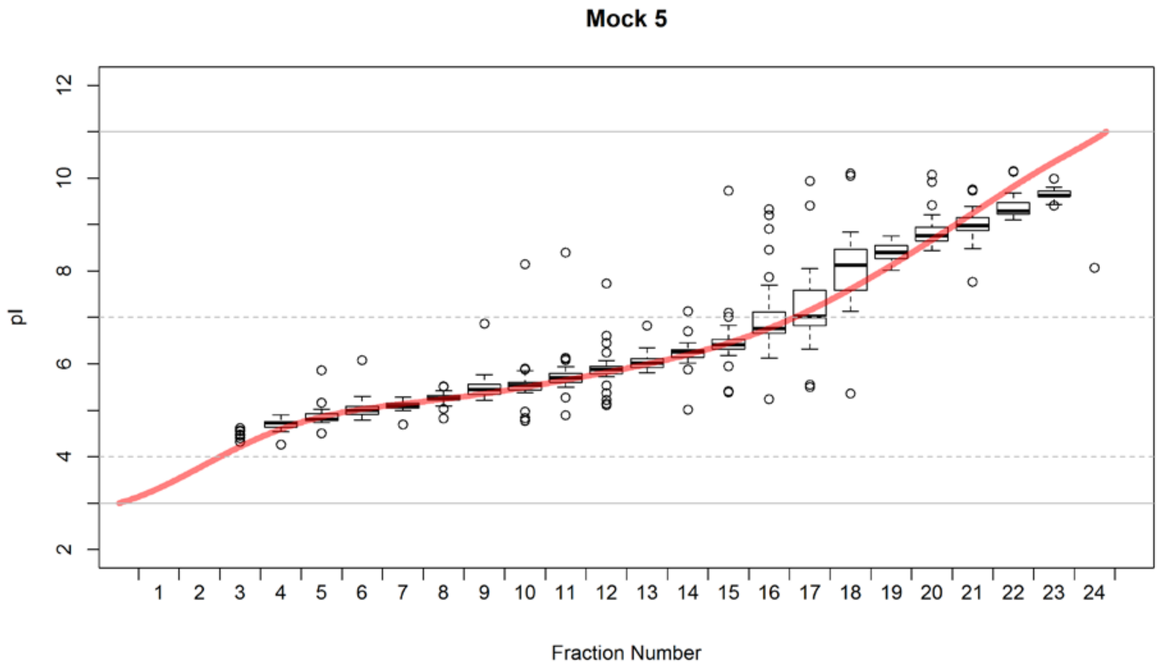
Mock 5
pI
2
4
6
8
10
12
1 2 3 4 5 6 7 8 9 10 11 12 13 14 15 16 17 18 19 20 21 22 23 24
Fraction Number

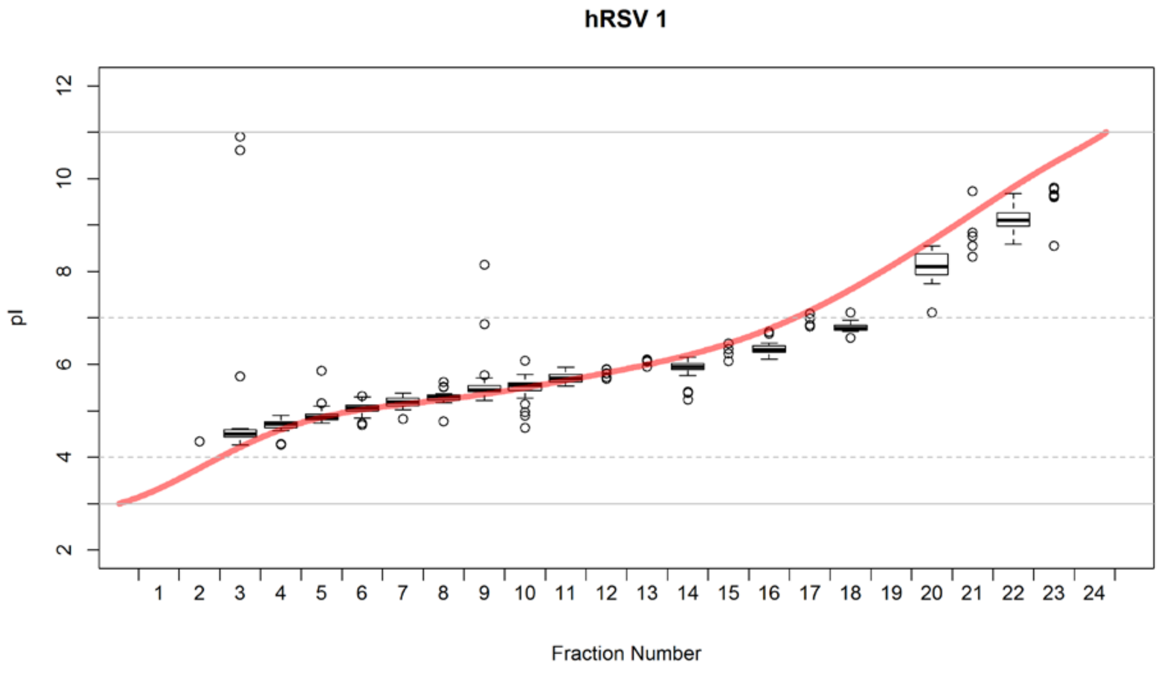
hRSV 1
pI
2
4
6
8
10
12
1 2 3 4 5 6 7 8 9 10 11 12 13 14 15 16 17 18 19 20 21 22 23 24
Fraction Number

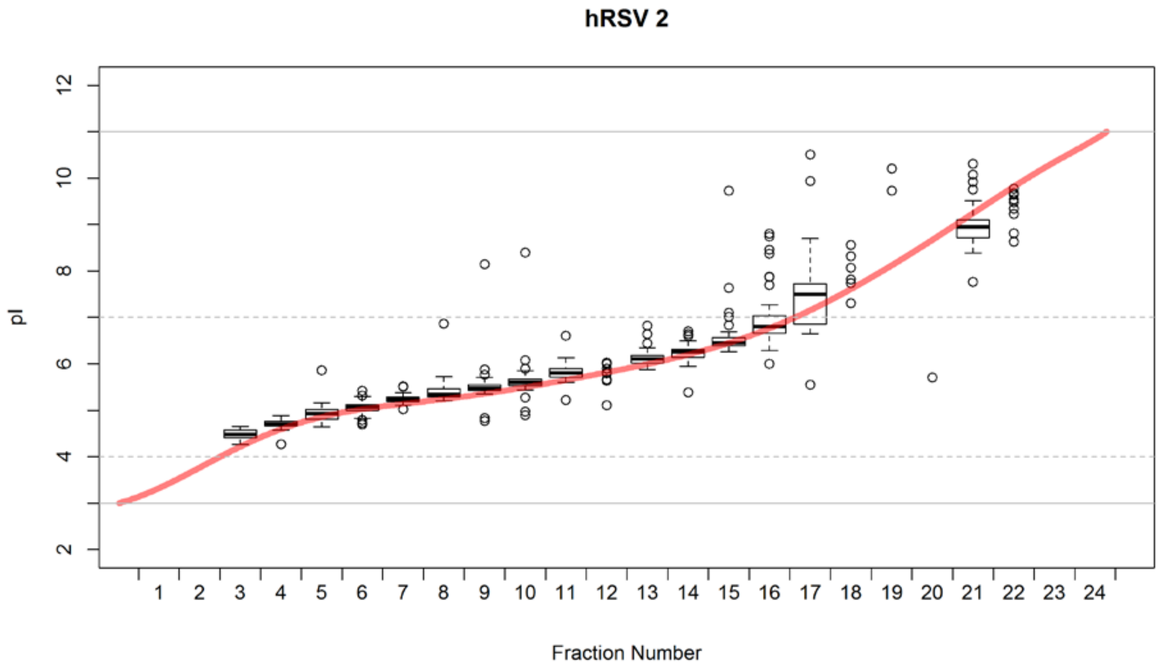
hRSV 2
pI
Fraction Number
2
4
6
8
10
12
1 2 3 4 5 6 7 8 9 10 11 12 13 14 15 16 17 18 19 20 21 22 23 24

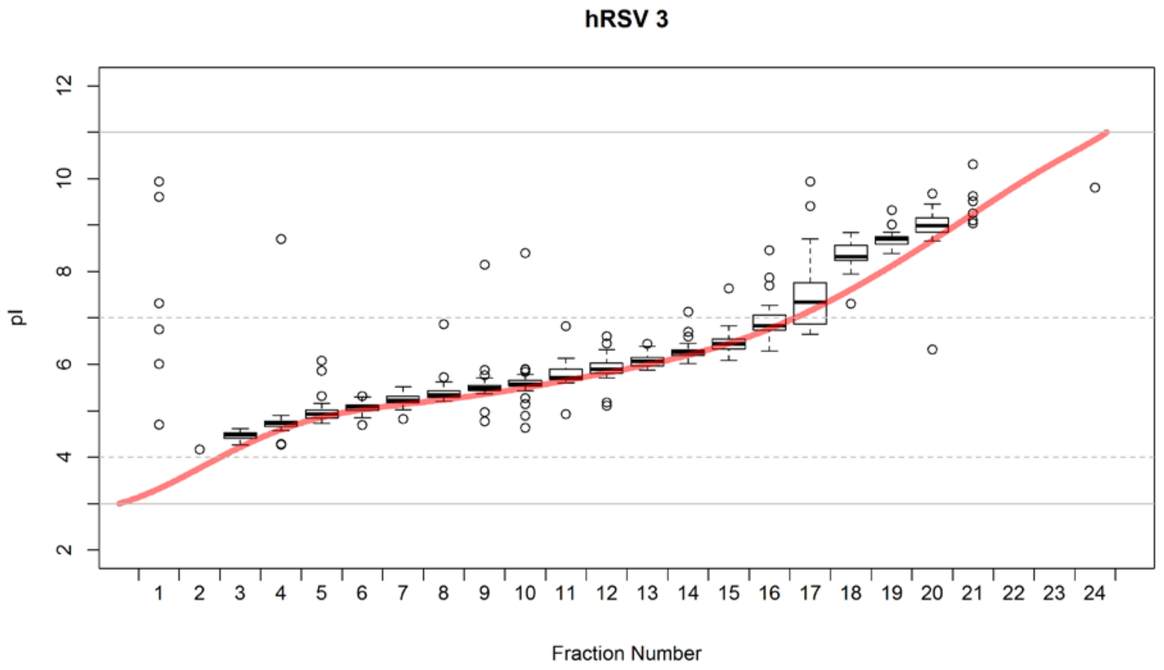
hRSV 3
pI
Fraction Number
2
4
6
8
10
12
1 2 3 4 5 6 7 8 9 10 11 12 13 14 15 16 17 18 19 20 21 22 23 24

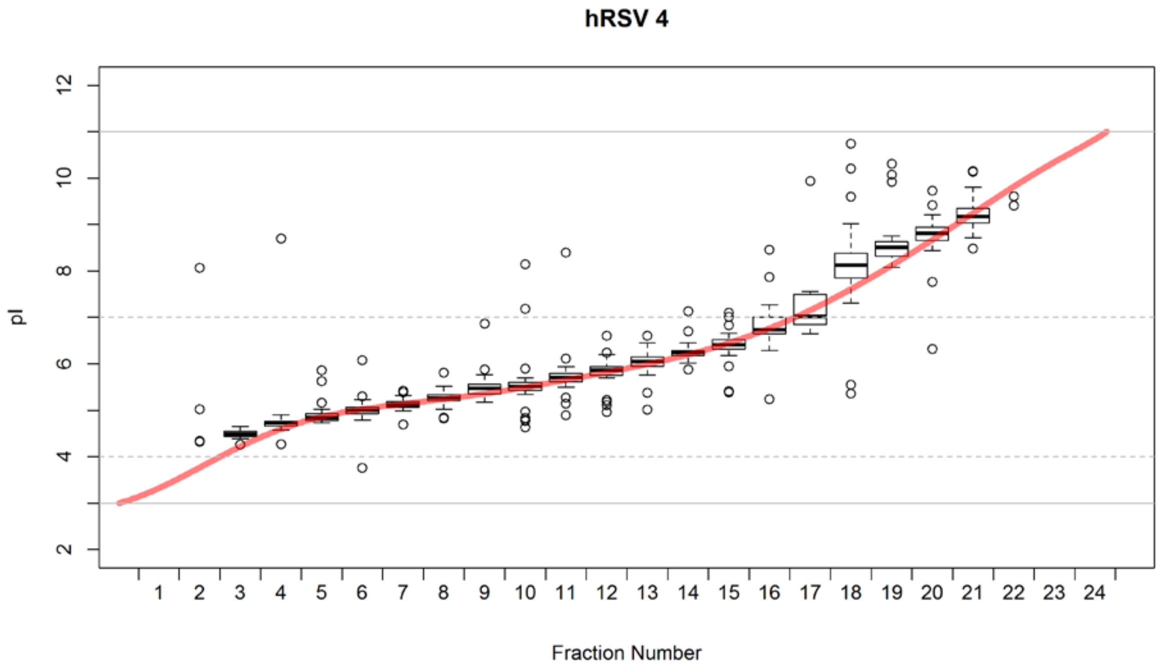

hRSV 4
pI
12
10
8
6
4
2
1 2 3 4 5 6 7 8 9 10 11 12 13 14 15 16 17 18 19 20 21 22 23 24
Fraction Number

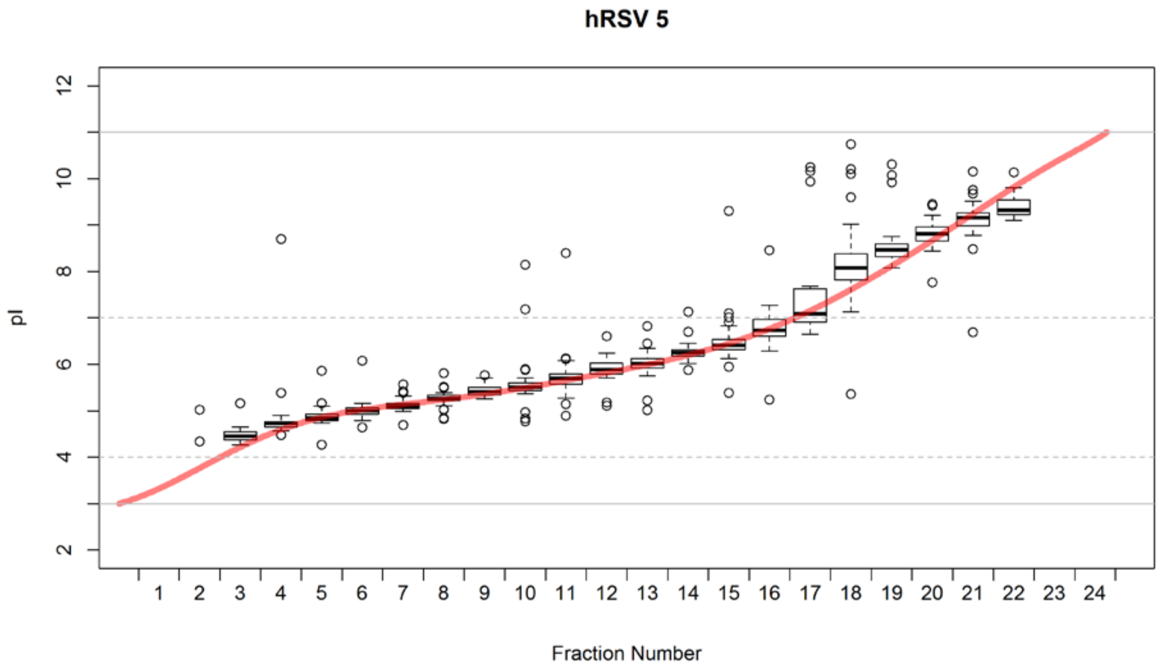

hRSV 5
pI
12
10
8
6
4
2
1 2 3 4 5 6 7 8 9 10 11 12 13 14 15 16 17 18 19 20 21 22 23 24
Fraction Number

Supplemental Fig. 2. **Traces for proteoform-specific peptides for the A) PSMB10, B) STAT1 and C) WARS protein groups.**

A) PSMB10

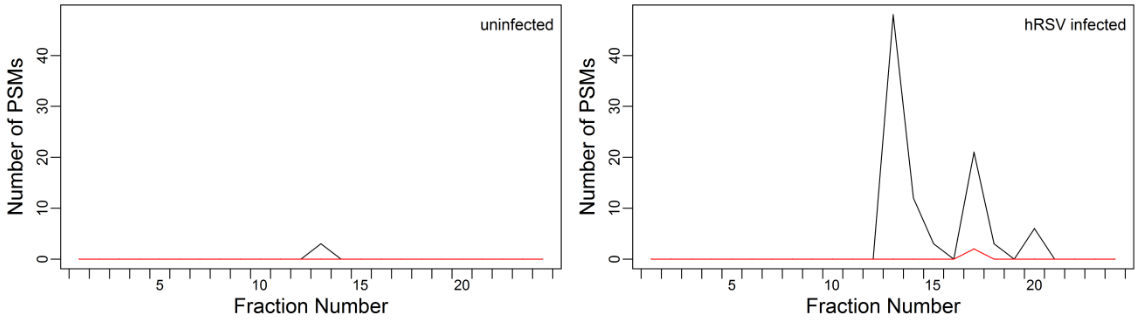

Traces for peptides assigned to the PSMB10 protein group in uninfected and hRSV-infected lysates (left and right, respectively). The ***RED*** traces represent PSMs for the N-terminal peptide that exclusively matched the precursor proteoform (peptide sequence MLKPALEPR exclusively matching P40306). The ***BLACK*** traces represent PSMs that matched both the precursor and processed PSMB10 proteoforms (P40306 and P40306 40-273 respectively).

B) STAT1

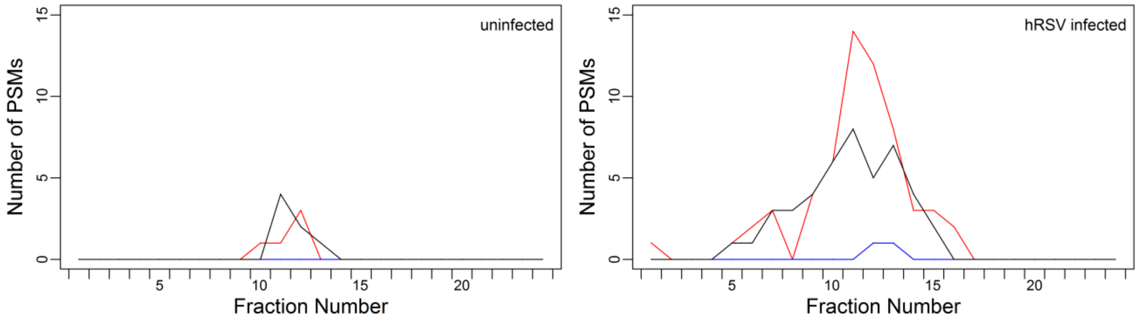

Traces for peptides assigned to the STAT1 protein group in uninfected and hRSV-infected lysates (left and right, respectively). The ***RED*** traces represent PSMs exclusively matching the Stat1-α proteoform (peptide sequences IVGSVEFDSMMNTV, LQTTDNLLPMSPEEFDEVSR and TELISVSEVHPSR exclusively matching proteoform sequence P42224) and the ***BLUE*** traces represent PSMs for the C-terminal peptide of Stat1-β (peptide sequence TELISVSEV matching Stat1-β proteoform sequences P42224-2 and J3KPM9). The ***BLACK*** traces represent an abundant peptide that matches both Stat1-α and Stat1-β proteoforms (peptide sequence FLEQVHQLYDDSFPMEIR matching proteoform sequences P42224, D2KFR9, P42224-2 and J3KPM9).

C) WARS

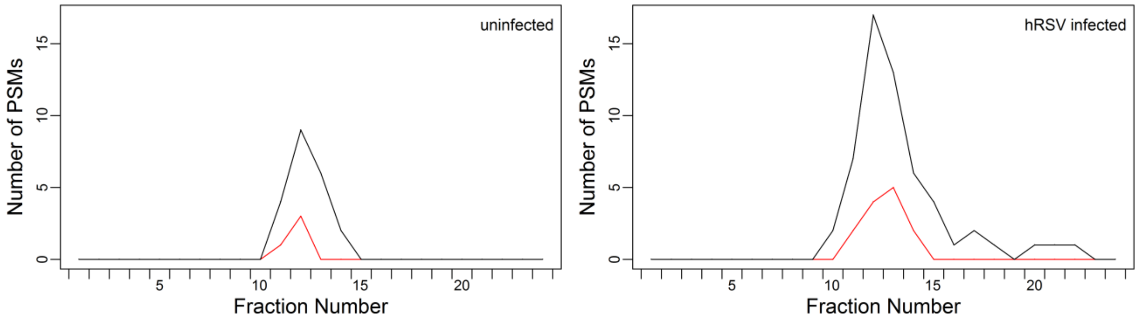

Traces for peptides assigned to the WARS protein group in uninfected and hRSV-infected lysates (left and right, respectively).The ***RED*** traces represent PSMs that exclusively matched the full length TrpRS sequence (peptide sequence AGNASKDEIDSAVK exclusively matching proteoform sequence P23381). The ***BLACK*** traces represent PSMs for an abundant shared peptide (ALIEVLQPLIAEHQAR) that matched the full length TrpRS proteoform (P23381), processed proteoforms T1-TrpRS and T2-TrpRS (P23381 71-471 and P23381 94-471, respectively) and the alternative splice form mini-TrpRS (P23381-2).

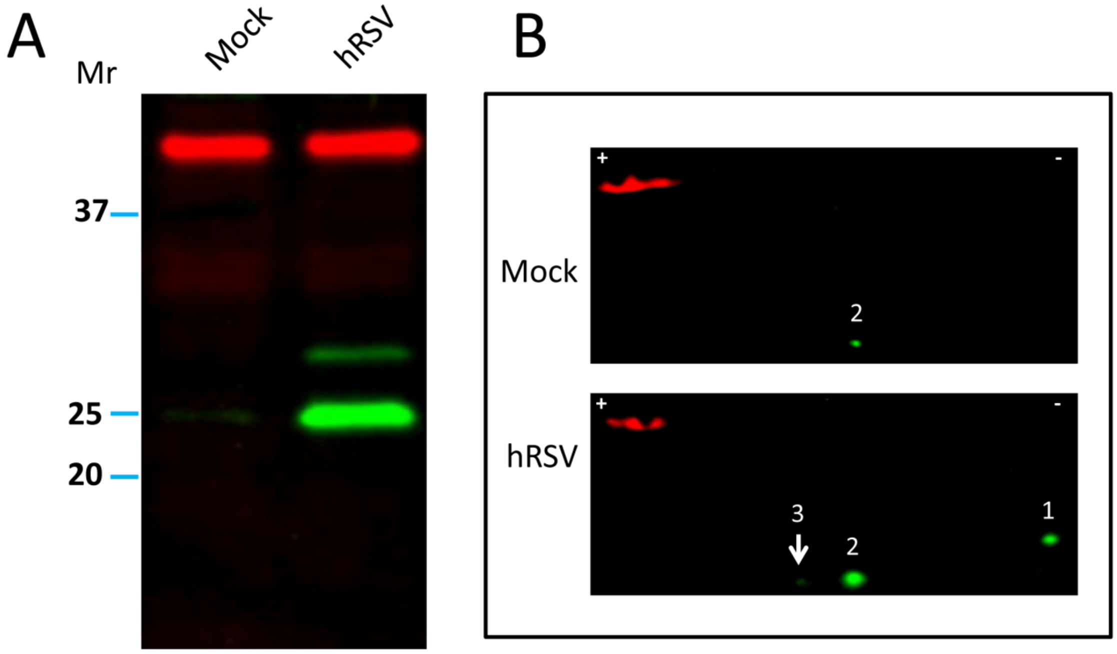

Supplemental Fig. 3. **Western blot characterization of PSMB10 proteoforms induced by hRSV infection of A549 cells.** (A) One-dimensional-Western blot uninfected (Mock) and hRSV-infected (hRSV) A549 whole cell lysates (10 µg). Green bands represent PSMB10 specific reactivity and the red band is β-actin specific reactivity. (B) Two-dimensional-Western blots of equal quantities (20 µg) of protein from lysates of Mock (top panel) and hRSV (bottom panel) infected A549 cells. Green spots correspond to the precursor (spot 1), activated (spot 2) and activated with an putative acidic PTM (arrowed spot 3) proteoforms of PSMB10. The red area of reactivity, corresponding to approximately 42 kDa, represents β-actin used as a loading control.

Supplemental Fig. 4. **Predicted focusing of MxA fragments.** Focusing is predicted for all MxA fragments generated by unspecific cleavage of the full length proteoform sequence P20591. Short fragments that do not contain at least one tryptic peptide were excluded from the analysis. Each black line represents an MxA fragment predicted to focus to the respective OGE fraction. For each OGE fraction, fragments are aligned to the full length MxA proteoform sequence (P20591) and are stacked according to the index of the N-terminal residue in the full length sequence, then according to the fragment length. MxA fragments containing the N-terminal region of MxA are predicted to focus to approximately fraction 4 to 9 whereas fragments containing the C-terminal region of MxA are predicted to focus to approximately fraction 14 to 21. The full length MxA proteoform sequence is predicted to focus to fraction 11.

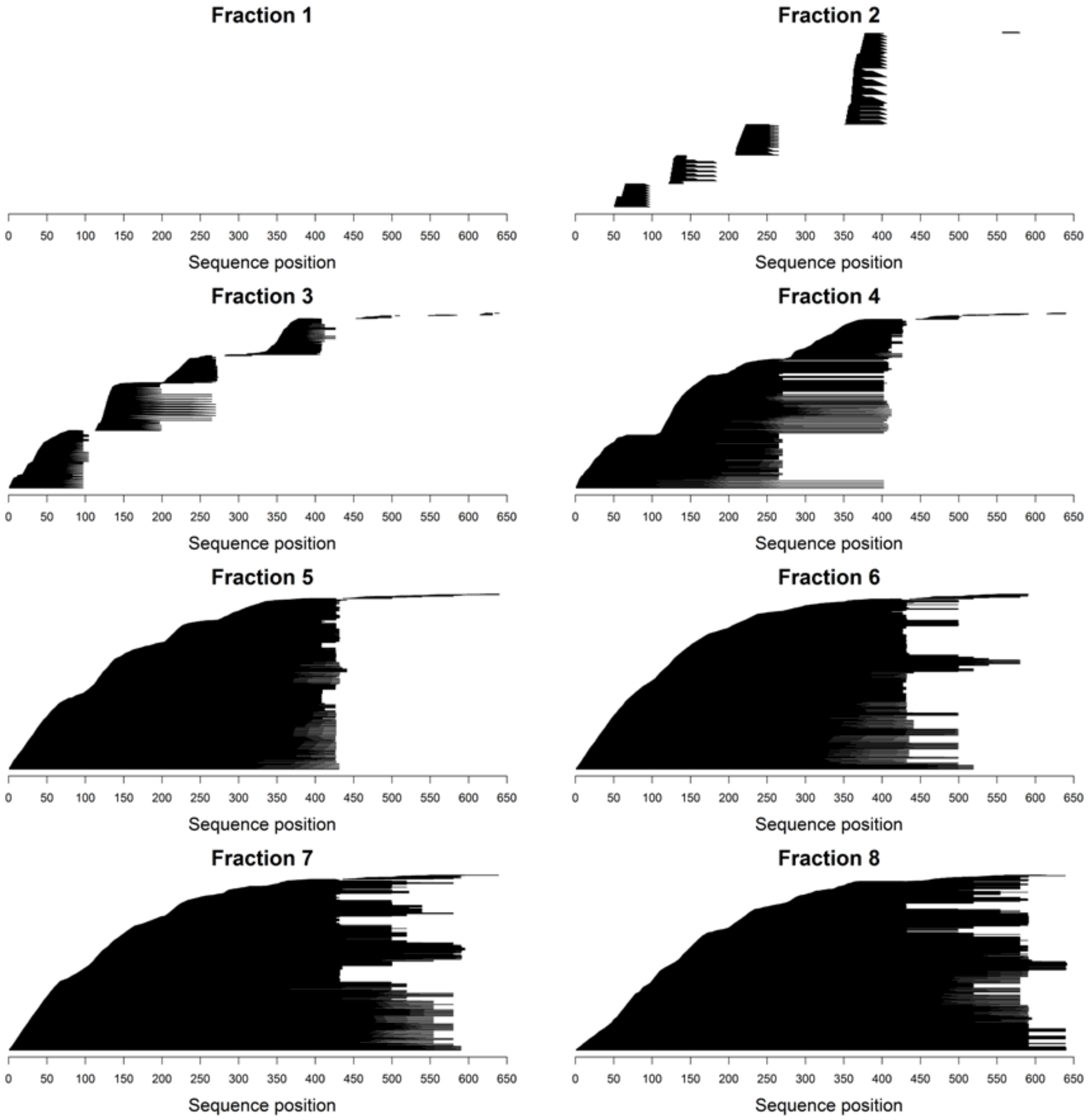

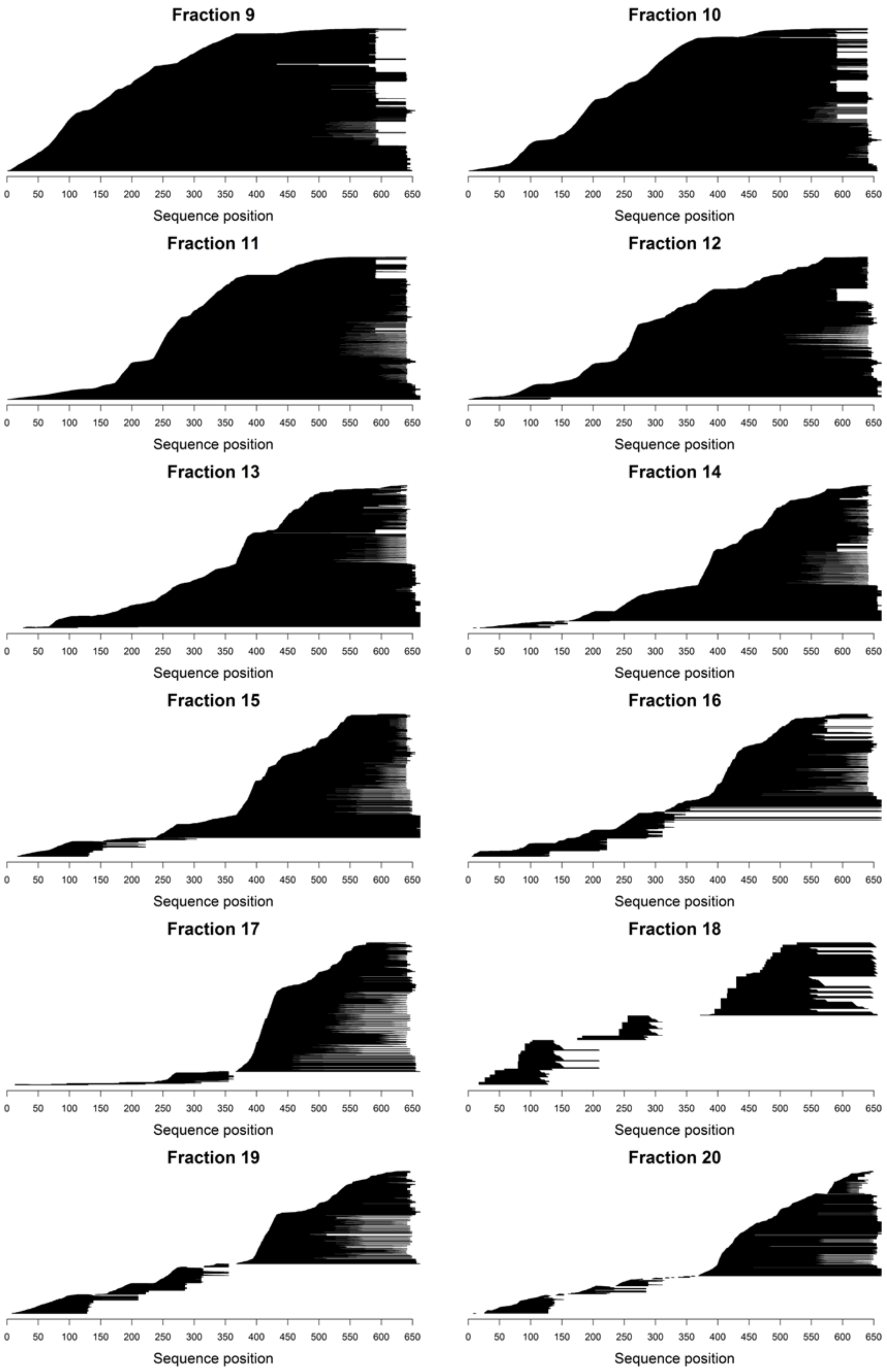

Fraction 9
Fraction 10
Fraction 11
Fraction 12
Fraction 13
Fraction 14
Fraction 15
Fraction 16
Fraction 17
Fraction 18
Fraction 19
Fraction 20
0 50 100 150 200 250 300 350 400 450 500 550 600 650
Sequence position

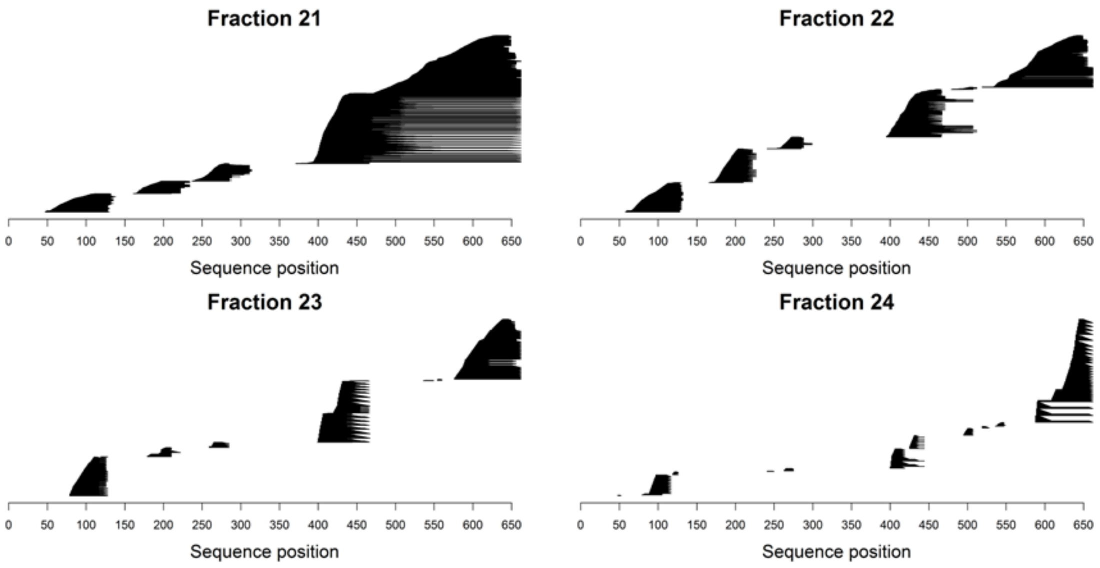

Fraction 21
0 50 100 150 200 250 300 350 400 450 500 550 600 650
Sequence position
Fraction 22
0 50 100 150 200 250 300 350 400 450 500 550 600 650
Sequence position
Fraction 23
0 50 100 150 200 250 300 350 400 450 500 550 600 650
Sequence position
Fraction 24
0 50 100 150 200 250 300 350 400 450 500 550 600 650
Sequence position

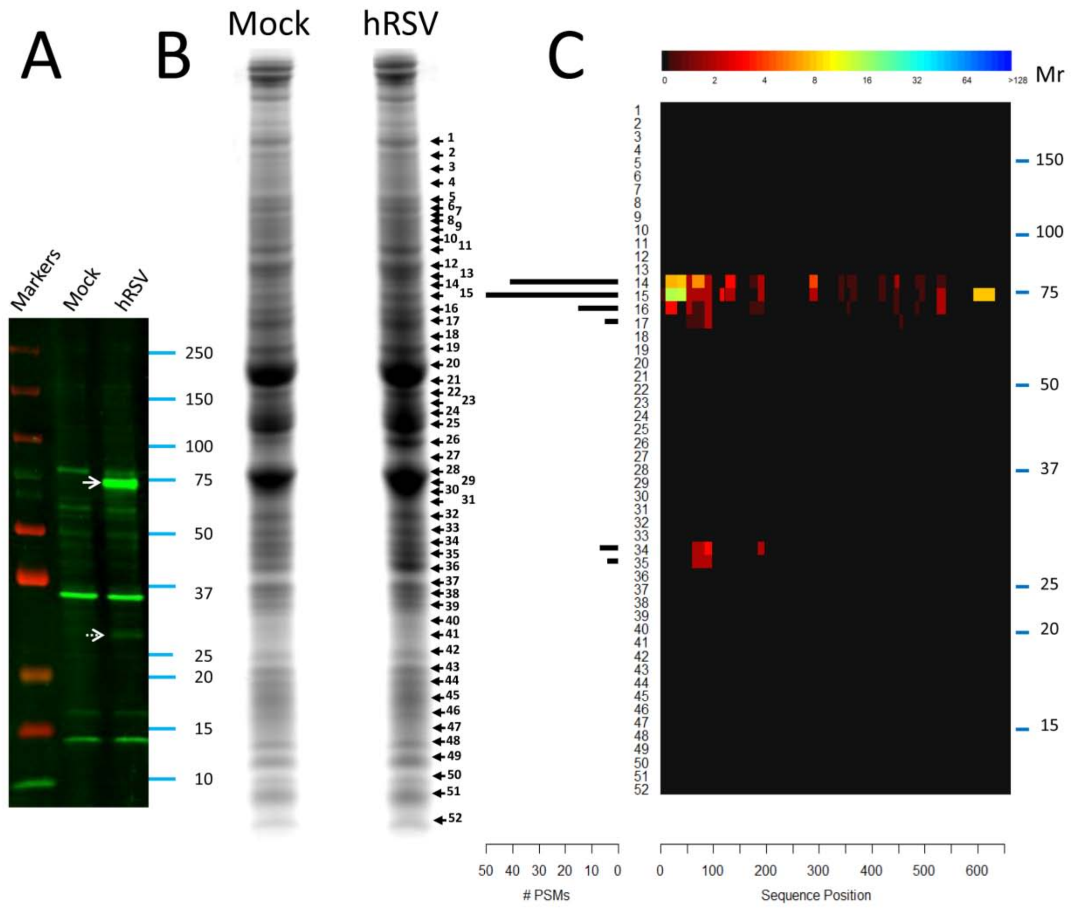

Supplemental Fig. 5. **Western blot and Protomap analyses of MxA proteoforms in unfractionated A549 whole cell lysates.** (A) One-dimensional-Western blot of uninfected (Mock) and hRSV-infected (hRSV) A549 whole cell lysates.The solid white arrow indicates the mobility of the full-length canonical MxA proteoform. The broken white arrow indicates the mobility of the 29 kDa MxA proteoform. (B) Stained 1D-SDS-PAGE gels of uninfected (Mock) and hRSV-infected (hRSV) A549 cell lysates which were sliced according to the numbered arrows. (C) Whereas in-gel digests of hRSV-infected lysate slices revealed PSMs for MxA indicated in the PSM histograms, uninfected lysates produced no PSMs for MxA. The sequence coverage heat map (right) represents the alignment of all PSMs identified in the in-gel slices at each position of the canonical MxA proteoform sequence (P20591).

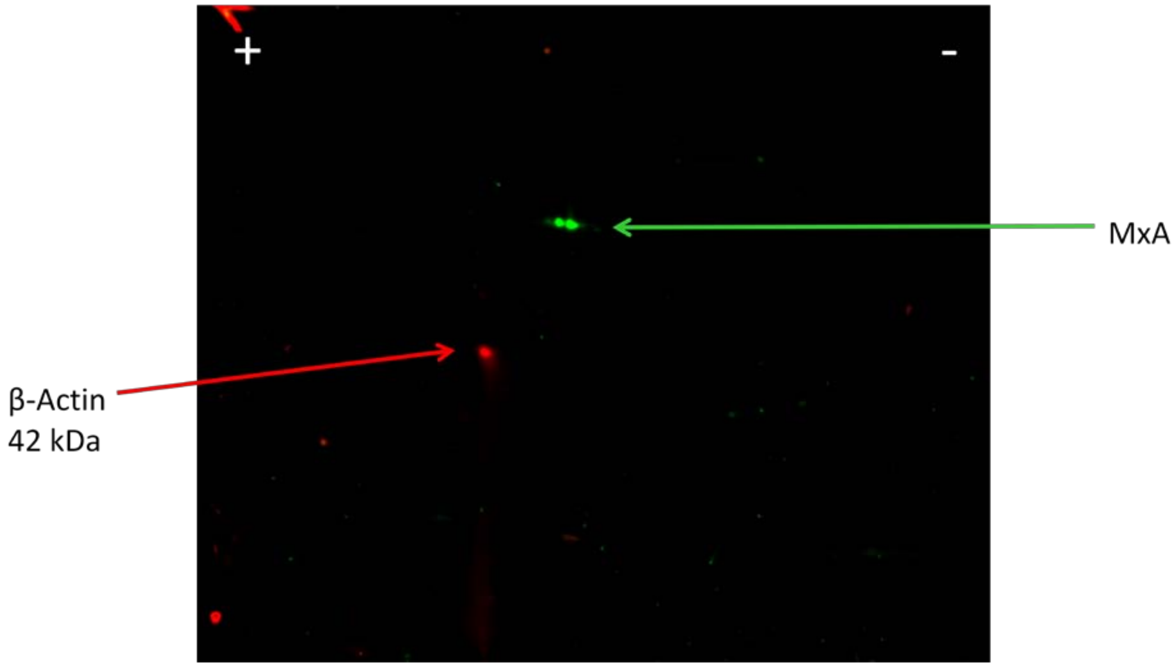

Supplemental Fig. 6. **Complete 2D--Western blot of an hRSV-Infected A549 cell lysate**. This blot was probed with an antibody to the N-terminal common to full length MxA and VarMxA.

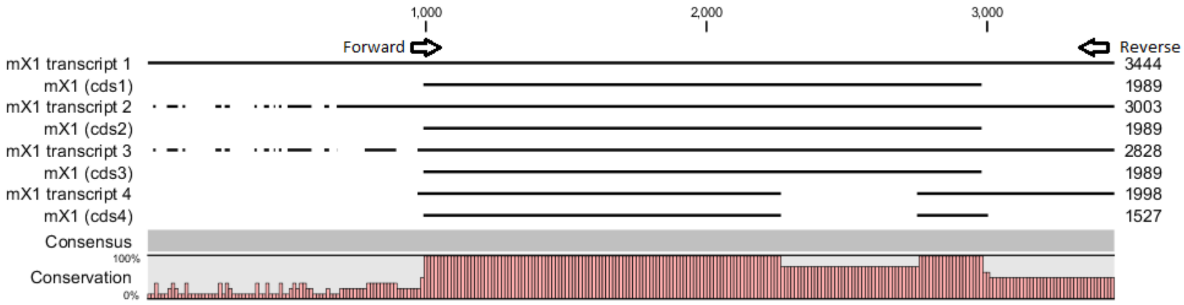

Supplemental Fig. 7. **Alignment of potential MX1 transcripts.** Reported transcript variants with accession numbers, NM_001144925.2, NM_002462.4, NM_001178046.2, NM_001282920.1, and their respective coding sequences (cds) were aligned. Oligonucleotide primers were designed to cover the entire cds indicated by arrows.

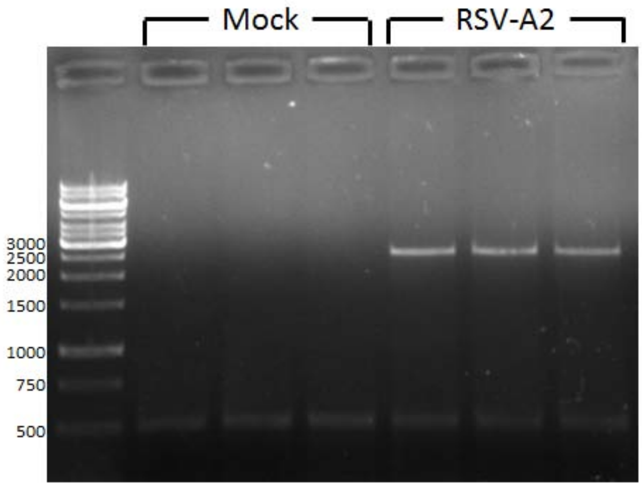

Supplemental Fig. 8. **PCR analysis of MX1 mRNA transcripts.** mRNA was extracted from mock and A2 hRSV infected A549 cells at 24hrs post infection. mRNA was reverse transcribed to cDNA and PCR was performed with MX1 specific primers.

```
LOCUS       mX1_(A549_RSV_induced)_CDS      2410 bp    DNA     linear   UNA
19-Sep-2014
DEFINITION
FEATURES             Location/Qualifiers
     CDS             20..2005
                     /label=mX1
ORIGIN
        1 TACTTTGCAA AGAAGGAAGA TGGTTGTTTC CGAAGTGGAC ATCGCAAAAG CTGATCCAGC
       61 TGCTGCATCC CACCCTCTAT TACTGAATGG AGATGCTACT GTGGCCCAGA AAAATCCAGG
      121 CTCGGTGGCT GAGAACAACC TGTGCAGCCA GTATGAGGAG AAGGTGCGCC CCTGCATCGA
      181 CCTCATTGAC TCCCTGCGGG CTCTAGGTGT GGAGCAGGAC CTGGCCCTGC CAGCCATCGC
      241 CGTCATCGGG GACCAGAGCT CGGGCAAGAG CTCCGTGTTG GAGGCACTGT CAGGAGTTGC
      301 CCTTCCCAGA GGCAGCGGGA TCGTGACCAG ATGCCCGCTG GTGCTGAAAC TGAAGAAACT
      361 TGTGAACGAA GATAAGTGGA GAGGCAAGGT CAGTTACCAG GACTACGAGA TTGAGATTTC
      421 GGATGCTTCA GAGGTAGAAA AGGAAATTAA TAAAGCCCAG AATGCCATCA CCGGGGAAGG
      481 AATGGGAATC AGTCATGAGC TAATCACCCT GGAGATCAGC TCCCGAGATG TCCCGGATCT
      541 GACTCTAATA GACCTTCCTG GCATAACCAG AGTGGCTGTG GGCAATCAGC CTGCTGACAT
      601 TGGGTATAAG ATCAAGACAC TCATCAAGAA GTACATCCAG AGGCAGGAGA CAATCAGCCT
      661 GGTGGTGGTC CCCAGTAATG TGGACATTGC CACCACAGAG GCTCTCAGCA TGGCCCAGGA
      721 GGTGGACCCC GAGGGAGACA GGACCATCGG AATCTTGACG AAGCCTGATC TGGTGGACAA
      781 AGGAACTGAA GACAAGGTTG TGGACGTGGT GCGGAACCTC GTGTTCCACC TGAAGAAGGG
      841 TTACATGATT GTCAAGTGCC GGGGCCAGCA GGAGATCCAG GACCAGCTGA GCCTGTCCGA
      901 AGCCCTGCAG AGAGAGAAGA TCTTCTTTGA GAACCACCCA TATTTCAGGG ATCTGCTGGA
      961 GGAAGGAAAG GCCACGGTTC CCTGCCTGGC AGAAAAACTT ACCAGCGAGC TCATCACACA
     1021 TATCTGTAAA TCTCTGCCCC TGTTAGAAAA TCAAATCAAG GAGACTCACC AGAGAATAAC
     1081 AGAGGAGCTA CAAAAGTATG GTGTCGACAT ACCGGAAGAC GAAAATGAAA AAATGTTCTT
     1141 CCTGATAGAT AAAATTAATG CCTTTAATCA GGACATCACT GCTCTCATGC AAGGAGAGGA
     1201 AACTGTAGGG GAGGAAGACA TTCGGCTGTT TACCAGACTC CGACACGAGT TCCACAAATG
     1261 GAGTACAATA ATTGAAAACA ATTTTCAAGA AGGCCATAAA ATTTTGAGTA GAAAAATCCA
     1321 GAAATTTGAA AATCAGTATC GTGGTAGAGA GCTGCCAGGC TTTGTGAATT ACAGGACATT
     1381 TGAGACAATC GTGAAACAGC AAATCAAGGC ACTGGAAGAG CCGGCTGTGG ATATGCTACA
     1441 CACCGTGACG GATATGGTCC GGCTTGCTTT CACAGATGTT TCGATAAAAA ATTTTGAAGA
     1501 GTTTTTTAAC CTCCACAGAA CCGCCAAGTC CAAAATTGAA GACATTAGAG CAGAACAAGA
     1561 GAGAGAAGGT GAGAAGCTGA TCCGCCTCCA CTTCCAGATG GAACAGATTG TCTACTGCCA
     1621 GGACCAGGTA TACAGGGGTG CATTGCAGAA GGTCAGAGAG AAGGAGCTGG AAGAAGAAAA
     1681 GAAGAAGAAA TCCTGGGATT TTGGGGCTTT CCAATCCAGC TCGGCAACAG ACTCTTCCAT
     1741 GGAGGAGATC TTTCAGCACC TGATGGCCTA TCACCAGGAG GCCAGCAAGC GCATCTCCAG
     1801 CCACATCCCT TTGATCATCC AGTTCTTCAT GCTCCAGACG TACGGCCAGC AGCTTCAGAA
     1861 GGCCATGCTG CAGCTCCTGC AGGACAAGGA CACCTACAGC TGGCTCCTGA AGGAGCGGAG
     1921 CGACACCAGC GACAAGCGGA AGTTCCTGAA GGAGCGGCTT GCACGGCTGA CGCAGGCTCG
     1981 GCGCCGGCTT GCCCAGTTCC CCGGTTAACC ACACTCTGTC CAGCCCCGTA GACGTGCACG
     2041 CACACTGTCT GCCCCCGTTC CCGGGTAGCC ACTGGACTGA CGACTTGAGT GCTCAGTAGT
     2101 CAGACTGGAT AGTCCGTCTC TGCTTATCCG TTAGCCGTGG TGATTTAGCA GGAAGCTGTG
     2161 AGAGCAGTTT GGTTTCTAGC ATGAAGACAG AGCCCCACCC TCAAATGCAC ATGAGCTGGC
     2221 GGGGATGAAG GATGCTGTCT TCGTACTGGG AAAGGGATTT TCAGCCCTCA GAATCGCTCC
     2281 ACCTTGCAGC TCTCCCCTTC TCTGTATTCC TAGAAACTGA CACATGCTGA ACATCACAGC
     2341 TTATTTCCTC ATTTTTATAA TGTCCCTTCA CAAACCCAGT GTTTTAGGAG CATGAGTGCC
     2401 GTGTGTGTGC
```

Supplemental Fig. 9. **DNA sequence of the PCR product as described in Supplemental Figs. 7 and 8.**

```
LOCUS       mX1_(A549_RSV_induced)_CDS_protein       662 aa
linear   UNA
FEATURES             Location/Qualifiers
ORIGIN
        1 MVVSEVDIAK ADPAAASHPL LLNGDATVAQ KNPGSVAENN LCSQYEEKVR PCIDLIDSLR
       61 ALGVEQDLAL PAIAVIGDQS SGKSSVLEAL SGVALPRGSG IVTRCPLVLK LKKLVNEDKW
      121 RGKVSYQDYE IEISDASEVE KEINKAQNAI TGEGMGISHE LITLEISSRD VPDLTLIDLP
      181 GITRVAVGNQ PADIGYKIKT LIKKYIQRQE TISLVVVPSN VDIATTEALS MAQEVDPEGD
      241 RTIGILTKPD LVDKGTEDKV VDVVRNLVFH LKKGYMIVKC RGQQEIQDQL SLSEALQREK
      301 IFFENHPYFR DLLEEGKATV PCLAEKLTSE LITHICKSLP LLENQIKETH QRITEELQKY
      361 GVDIPEDENE KMFFLIDKIN AFNQDITALM QGEETVGEED IRLFTRLRHE FHKWSTIIEN
      421 NFQEGHKILS RKIQKFENQY RGRELPGFVN YRTFETIVKQ QIKALEEPAV DMLHTVTDMV
      481 RLAFTDVSIK NFEEFFNLHR TAKSKIEDIR AEQEREGEKL IRLHFQMEQI VYCQDQVYRG
      541 ALQKVREKEL EEEKKKKSWD FGAFQSSSAT DSSMEEIFQH LMAYHQEASK RISSHIPLII
      601 QFFMLQTYGQ QLQKAMLQLL QDKDTYSWLL KERSDTSDKR KFLKERLARL TQARRRLAQF
      661 PG
```

Supplemental Fig.10. **MxA protein sequence**. Complete amino acid sequence translated from the DNA sequence presented in Supplemental Fig. 9.
